# Structural Comparison of Sulfonamide-Based Derivatives That Can Improve Anti-Coagulation Properties of Metformin

**DOI:** 10.3390/ijms23084132

**Published:** 2022-04-08

**Authors:** Agnieszka Zajda, Joanna Sikora, Kristiina M. Huttunen, Magdalena Markowicz-Piasecka

**Affiliations:** 1 Laboratory of Bioanalysis, Department of Pharmaceutical Chemistry, Drug Analysis and Radiopharmacy, Medical University of Lodz, ul. Muszyńskiego1, 90-151 Lodz, Poland; agnieszkazajda95@gmail.com; 2 Department of Bioinorganic Chemistry, Medical University of Lodz, ul. Muszyńskiego1, 90-151 Lodz, Poland; joanna.sikora@umed.lodz.pl; 3 School of Pharmacy, Faculty of Health Sciences, University of Eastern Finland, Yliopistonranta 1C, P.O. Box 1627, 70211 Kuopio, Finland; kristiina.huttunen@uef.fi

**Keywords:** biguanides, coagulation, haemostasis, metformin

## Abstract

Due to its high efficiency, good safety profile, and potential cardio-protective properties, metformin, a dimethyl biguanide, is the first-line medication in antihyperglycemic treatment for type 2 diabetic patients. The aim of our present study was to assess the effects of eight new sulfonamide-based derivatives of metformin on selected plasma parameters and vascular hemostasis, as well as on endothelial and smooth muscle cell function. The compounds with an alkyl chain (**1**–**3**), trifluoromethyl substituent (**4**), or acetyl group (**5**) significantly elevated glucose utilization in human umbilical endothelial cells (HUVECs), similarly to metformin. Our novel findings showed that metformin analogues **1**–**3** presented the most beneficial properties because of their greatest safety profile in the WST-1 cell viability assay, which was also proved in the further HUVEC integrity studies using RTCA DP. Compounds **1**–**3** did not affect either HUVEC or aortal smooth muscle cell (AoSMC) viability up to 3.0 mM. Importantly, these compounds beneficially affected some of the coagulation parameters, including factor X and antithrombin III activity. In contrast to the above-mentioned metformin analogues, derivatives **4** and **5** exerted more profound anticoagulation effects; however, they were also more cytotoxic towards HUVECs, as IC_50_ values were 1.0–1.5 mM. In conclusion, the chemical modification of a metformin scaffold into sulfonamides possessing alkyl substituents results in the formation of novel derivatives with potential bi-directional activity including anti-hyperglycemic properties and highly desirable anti-coagulant activity.

## 1. Introduction

De novo drug development is a laborious and resource- and time-consuming task. Generally, it takes at least 13 years to discover a new drug, and the entire cost ranges from one to three billion dollars. Moreover, the drug design process is often complicated by safety issues, such as high toxicity and efficacy. Accordingly, only ca. 10% of novel compounds which enter first phase clinical trials are accepted to further clinical studies [1,2]. One alternative is drug repurposing, also named drug repositioning or drug reprofiling, for new indications. Although this is not a novel process, it has become an attractive alternative for traditional drug development in recent years. The aim of drug repurposing is to identify a new indication for drugs which are already approved. One advantage of this process is that as the repurposing candidates have established preclinical and clinical profiles (pharmacokinetic, pharmacodynamic and toxicity), they can immediately accede to late-stage clinical trials. The new targets of drug repositioning might be identified in several ways. The drug’s molecular purpose can demonstrate a beneficial effect in a different medical condition than originally indicated, or the drug may act via unrecognized targets associated with various diseases. The particular compound may also demonstrate additional and unintended benefits during clinical observation [3,4,5,6].

The sulfonamides represent a large group of well-studied drugs which possess a wide range of antibacterial, antiviral, and antifungal activities, as well as various antimalarial, anti-inflammatory, hypoglycemic, anti-cancer properties, among others. As such, they can be used in a wide range of disease entities. The versatile structure of sulfonamides and the wide range of their activities have contributed to increased interest in repurposing old drugs. The design and synthesis of novel sulfonamide derivatives with beneficial effects are of particular concern [7].

Recent scientific interest has also been focused on the advantageous effects of sulfonamide derivatives on the cardiovascular system and hemostasis as a result of their regulation of vascular tone and influence on the renin angiotensin system, endothelin receptor, and lipid metabolism. The ability of newly-designed pyrrolidine-sulfonamides to suppress transient receptor potential vanilloid-4 (TRPV4) allows them not only regulate Ca^2+^/Na^+^ inflow into vascular endothelial cells in lungs, but also to control vascular tone and presumably reduce the risk of heart failure (HF) [7,8,9,10,11]. The development of atherosclerosis and hypertension is accelerated by increased secretion of fatty acid-binding protein (FABP4) and retinol-binding protein (RBP-4). One potential therapeutic strategy in the treatment of atherosclerosis or other cardiovascular diseases is the use of naphthalene-1-sulfonamide derivatives, which are selective FABP4 inhibitors [12,13]. Structural modifications of naphthalene-1-sulfonamide derivatives have been found to inhibit lipolysis, promote lipogenesis, and then reduce glucose and serum lipid levels in obese diabetic mice [14,15]. Another study showed that sulfonamide-pyrrolidinones are also potent and selective inhibitors of factor Xa, which can lead to prevention of thrombin production [16].

Another sulfonamide derivative, rosuvastatin, also has advantageous effects on lipid metabolism. This is commonly used in the treatment of hypercholesterolemia, and has already shown a significant reduction of serum RBP-4 level at a low dose of 2.5 mg/day [17]. The renin-angiotensin-aldosterone (RAA) and endothelin system play important roles in the pathogenesis of hypertension. Activation of endothelin-1 (ET_1_) secretion and angiotensin I (AT I) to angiotensin II conversion (ATII) induces blood vessel constriction and promotes the development of vessel change, which restricts blood flow and increases blood pressure. For instance, bosentan, a dual endothelin (ET_A_/ET_B_) receptor antagonist with a sulfonamide structure, is currently used in the treatment of pulmonary artery hypertension (PAH); however, its potential effectiveness has not been confirmed in all trials, and, importantly, this effect may depend on the duration of treatment in patients with heart failure. There are currently insufficient data on the long-term effect of ET_A_/ET_B_ receptor antagonism induced by bosentan in hypertension [18,19].

In addition, *n*-butyl, methyl, ethoxymethyl, or methoxymethyl substitution of sulfonamide derivatives of benzimidazole have also been found to offer potential as angiotensin II subtype 1 (AT_1_) and endothelin subtype A (ET_A_) antagonists [20,21]. There is also some evidence that biphenyl sulfonamide with an unsubstituted 1,2,4-triazole moiety may be a strong and selective angiotensin II subtype 2 (AT_2_) receptor agonist. This compound has been found to exert antihypertension activity on systolic arterial pressure in mice [22].

Metformin is the first-line oral drug used for initial monotherapy in patients with type 2 diabetes mellitus (T2DM). In the later stages of treatment, metformin is used in co-administration with insulin and other hypoglycemic agents. Its favorable action includes reduction of hepatic glucose production and lowering of insulin resistance, as well as having an advantageous influence on lipid metabolism and cardioprotection activity [23,24]. Metformin also demonstrates a favorable influence on vascular cells by significantly reducing the expression of intercellular adhesion molecules, such as the soluble vascular cell adhesion molecule (sVCAM), soluble intercellular adhesion molecule (sICAM), and von Willebrand factor (vWF), and by inhibiting the adhesion of monocytes to human endothelial cells and preventing the formation of foam cells [25,26,27,28]. In addition, recent studies indicate that metformin possesses the ability to increase the release of tissue plasminogen activator (t-PA) from human umbilical endothelial cells (HUVECs) and to decrease procoagulant activity of tissue factor (TF) [28,29]. Metformin has also been found to exert beneficial effects on platelet function. For instance, Xin et al. reported that metformin has the ability to suppress mtDNA release, leading to the inhibition of platelet activation through a C-type lectin receptor (DC-SIGN)-dependent pathway, thus preventing both venous and arterial thrombosis [30].

These multidirectional effects of metformin therapy have encouraged researchers to modify the structure of biguanide to enhance its potentially favorable activity. In the field of cardiovascular diseases and hemostasis, it was found that sulfonamide-based derivatives of metformin positively affect plasma hemostasis, including clot formation, stabilization, fibrinolysis, and also platelet activity [31]. In addition, Xin et al. found that introduction of a sulfonamide group to a biguanide derivative can inhibit the formation of platelet thrombus by decreasing platelet aggregation, adhesion, and clot retraction. Furthermore, a newly-synthesized N-trifluoromethanesulfonyl biguanide derivative was also found to hinder the activation of integrin GPIIb/IIIa activation and type-1 transmembrane protein (CD62P) expression [32]. These findings have spurred interest in sulfa-drugs, including the biguanide modification into sulfonamides, and these compounds have become promising candidates for the repurposing of old drugs.

**Figure 1:**
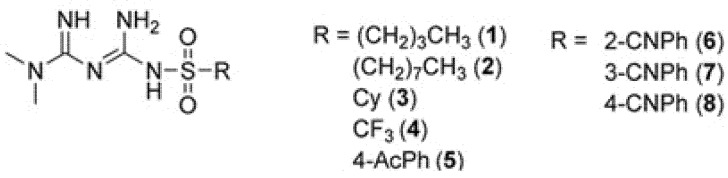
Structure of examined biguanides. Explanatory notes: Ac—acetyl, CN—cyano, Cy—cyclohexyl, Ph—phenyl.

The aim of the present study was to compare the effects of metformin derivatives, consisting of a biguanide scaffold with various types of substituted sulfonamide moiety, on plasma hemostasis and endothelial function, thus gaining a better understanding of its activity in this regard. The effects of eight novel sulfonamide-based metformin derivatives (Figure 1) were assessed with regard to their glucose utilization and basic coagulation plasma hemostasis parameters. The studies included also more advanced coagulation parameters, such as the activity of coagulation Xa factor and antithrombin III (AT). In further studies, the examined compounds were screened for the overall potential of clot formation and fibrinolysis (CL-test). The second part of the study examined their effects on the viability, integrity, and morphology of HUVEC and human aortal smooth muscle cells (AoSMC). Following this, the study examined the impact of these novel metformin derivatives on the release of (t-PA) from human endothelial cells. Our findings showed that the chemical modification of the metformin scaffold into sulfonamides containing trifluoromethyl and an acetyl group leads to the formation of potential dual-action drug candidates with glucose-lowering properties and highly desirable anti-coagulant activity.

## 2. Results

### 2.1. Intracellular Glucose Uptake

**Figure 2:**
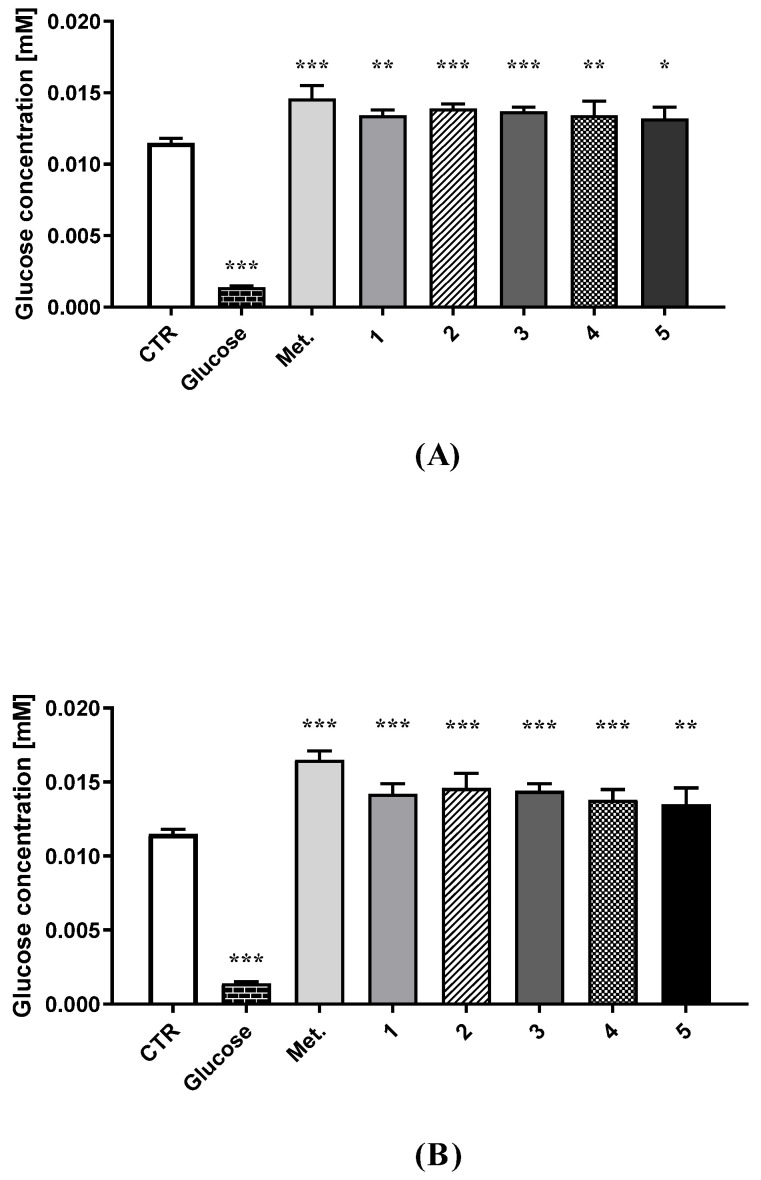
Effects of metformin and biguanide analogues **1**–**5** at the concentration of 0.1 (**A**) and 0.3 mM (**B**), and D-glucose at the concentration of 0.1 mM on the 2-NBDG uptake in HUVEC cells. The results are presented as mean ± SD, *n* = 4–6, and an asterisk denotes the significant differences between control and cells treated with glucose, metformin, or studied compounds. * *p* < 0.05; ** *p* < 0.01; *** *p* < 0.001.

The effects of the examined sulfonamides (**1**–**8**) on glucose uptake in HUVEC cells were assessed using a fluorescent glucose analogue (2-NBDG). In these experiments, the cells were treated with compounds **1**–**8** at 0.1 and 0.3 mM for 24 h, followed by two-hour glucose starvation, and subsequent incubation with insulin and 2-NBDG (Figure 2 and Figure S1, Table S1, Supplementary Materials). D-glucose was used as a negative control, which significantly inhibited the uptake of 2-NBDG (0.0014 ± 0.0001 mM versus 0.0115 ± 0.0003 mM for control, *p* < 0.001). A significant increase in 2-NBDG uptake was observed in the case of the reference drug, metformin, at both tested concentrations (0.0146 ± 0.0009 mM at 0.1 mM, and 0.0165 ± 0.0006 mM at 0.3 mM, *p* < 0.001). Importantly, compounds **1**–**5** were found to exert comparable effects to the parent drug, and profoundly elevated intracellular uptake of fluorescently labelled glucose (Figure 2). Comparable effects were reported for compounds **6**, **7**, and **8** at 0.1 mM. These compounds possessing cyano substituents in the aromatic ring slightly increased glucose uptake; however, the changes were statistically insignificant (Figure S1, Table S1, Supplementary Materials). Compounds **7** and **8**, with a CN group in the meta and para positions, at 0.3 mM contributed to the increased uptake of glucose in HUVEC cells (*p* < 0.05).

### 2.2. Viability of HUVEC and AoSMC Cells

The effects of compounds **1**–**8** on the viability of HUVEC and AoSMC cells were determined using WST-1 assay. The cells were incubated with the test compounds at various concentrations ranging from 0.006 mM to 5.0 mM. The results are presented in Table 1.

Among the tested compounds, derivative **2** with an *n*-octyl tail appeared to have the lowest effect on the viability of HUVECs, since, within the concentration range 0.006–5 mM, HUVEC viability decreased to 32.1%. Compounds **1** and **3** were also characterized by a relatively good safety profile, as their IC_50_ values were 3.643 ± 0.26 and 3.506 ± 0.24 mM, respectively. The obtained results indicate that compounds **1**–**5** inhibited HUVECs growth at the level of 24.15%–30.39% at the concentrations of 0.3 mM (Table S2, Supplementary Materials). For instance, compound **1** and **3** at the concentration of 0.3 mM contributed to the decrease of HUVECs viability by approximately 28.96% and 24.15%, respectively. The viability of HUVECs after 24-h stimulation with compound **2** at 0.3 mM was 71.48 ± 3.12% vs. 101.87 ± 2.71% for control. Based on the obtained results from the viability assay, we can conclude that compounds **4** and **5** exerted more toxic influence on HUVEC cell growth up, and their IC_50_ values were 1.038 ± 0.15 and 1.589 ± 0.13 mM, respectively. Compounds **6**–**8**, with a cyano substituent in the aromatic ring, were much more toxic towards HUVECs, with IC_50_ values ranging from 0.30 to 0.79 mM. In this subgroup of biguanides, compound **8** presented the highest IC_50_ value, which suggests that the para position of the CN group in the aromatic ring could be associated with greater safety, expressed as higher cell viability.

Test compounds **1**–**8** seemed to have a much lower effect on the viability of AoSMC, as IC_50_ values in the concentration range of 0.006–5 mM could not be determined for a few compounds (Table 1). However, for the remaining compounds, the IC_50_ values were significantly higher than their respective values for HUVEC cells. For instance, the compounds with the cyano group in the aromatic ring demonstrated IC_50_ values of 1.57–1.89 mM for AoSMC cells.

**Figure 3:**
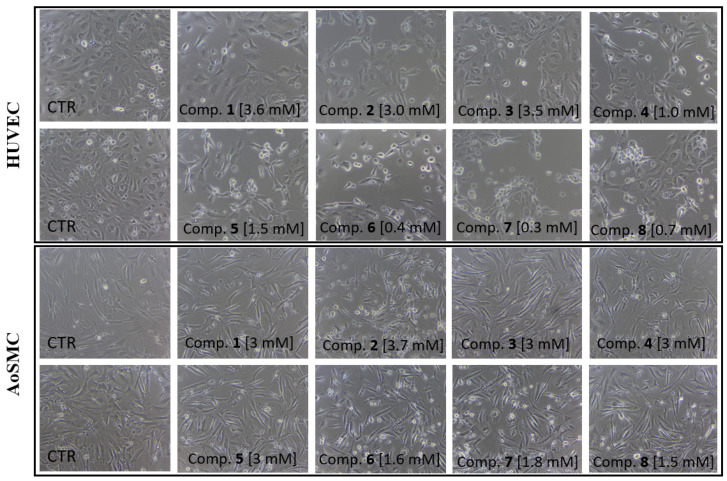
Effect of compounds **1**–**8** on HUVEC and AoSMC viability and morphology after 24-h incubation. Cells were cultured without (control, CTR) and in the presence of compounds **1**–**8** at a concentration of 0.006–5.0 mM. Representative cell images are shown for concentrations corresponding to IC50 values or 3 mM (100-fold magnification). Metformin was found to not affect the viability and morphology of HUVEC and AoSMC cells in our previous study [28].

The effects of compounds **1**–**8** on HUVEC and AoSMC cells were also determined using light and phase-contrast microscopy. Representative images of cells treated with compounds at their IC_50_ value or at 3.0 mM are depicted in Figure 3. In HUVECs, treatment with the examined biguanides resulted in the formation of rounded and shrunken cells; in addition, in the case of compounds **4**–**8**, elongation of cell bodies could be observed. The tested compounds appeared to have less influence on the morphology of AoSMC cells, manifested mainly by an increase in the number of round cells.

### 2.3. HUVEC Cell Integrity

**Figure 4:**
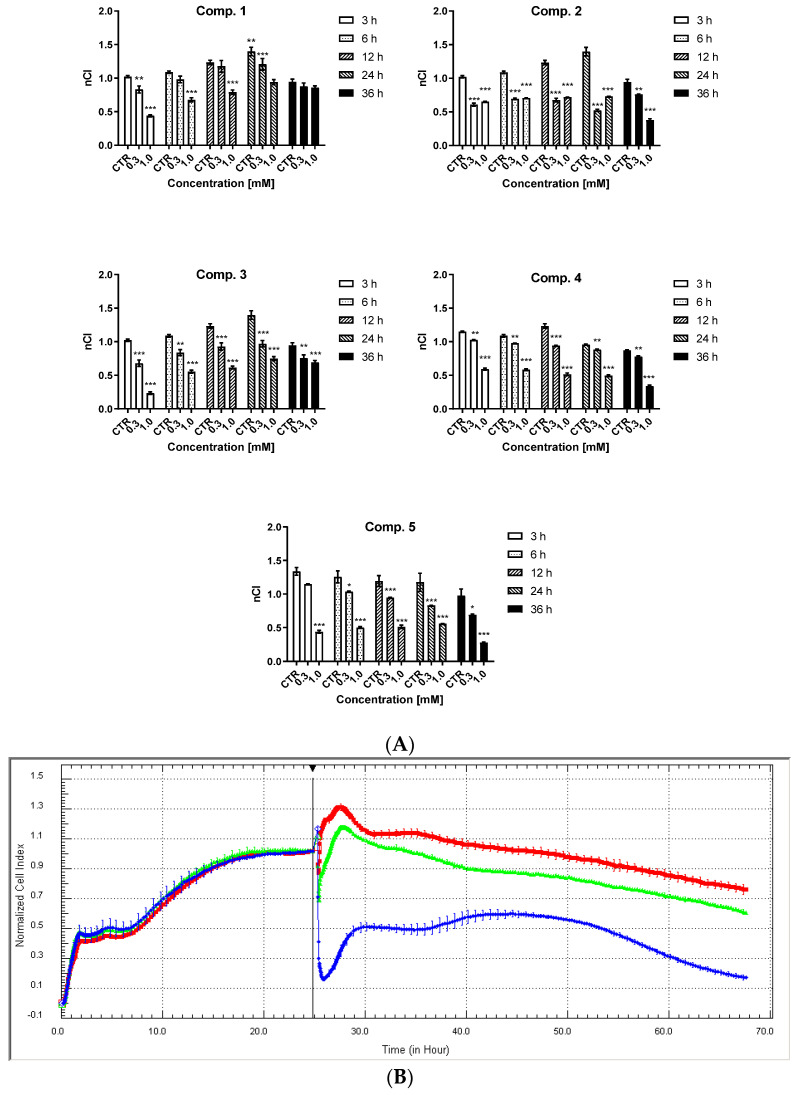
The effects of compounds **1**–**5** on the integrity of HUVEC analyzed in the RTCA-DP system. (**A**) The effect of the exposure of compounds **1**–**5** on normalized Cell Index (nCI) at selected time points (3, 6, 12, 24, and 36 h). The results are presented as mean ± SD, *n* = 4–5. An asterisk denotes a significant difference between sample treated with biguanide and control sample; * *p* < 0.05; ** *p* < 0.01; *** *p* < 0.001. (**B**) The effects of compound **5** on the barrier properties of HUVECs. The picture presents representative plots of one experiment conducted in duplicates (the results are presented as a mean (solid line) ± standard deviation). For the statistical analysis, three independent experiments were conducted. Red line—control (unstimulated cells); green line—compounds at the concentration of 0.3 mM; navy blue line—1.0 mM.

In HUVECs, the results of 36-h stimulation with the test compounds at concentrations of 0.3 and 1.0 mM are presented in Figure 4 (compounds **1**–**5**), Figure S2 (compounds **6**–**8**), and Table S3 (compounds **1**–**8**) (Supplementary Materials). Cell integrity and shape, expressed by normalized cellular index (nCI), were affected by the structure and concentration of the examined biguanides. All studied compounds caused a profoundly greater reduction in nCI at a concentration of 1.0 mM than 0.3 mM over the entire time of observation.

Statistical analysis indicated that compound **1**, with an *n*-butyl chain attached to a sulfonamide moiety, exerted the least effect on the function of HUVEC cells: no statistically significant reduction of nCI was noticed after 36 h of stimulation (Table S3). The compounds with CN substituents in the aromatic ring (**6**–**8**) demonstrated the most unfavorable effect on HUVEC barrier properties; when administered at 1.0 mM, compounds **6**–**8** decreased nCI drastically, which was close to zero, while the other compounds reduced the integrity of endothelial cells by 40–60% depending on the time point of the analysis.

Metformin was not found to affect the integrity and adherence of HUVEC cells over the entire concentration range (0.006–1.5 mM) in our previous study [28]. For instance, after 24 h of co-stimulation with metformin at 1.5 mM, nCI was 0.95 ± 0.08 versus control 1.01 ± 0.07 [28].

### 2.4. Endothelial Cell Migration

Based on the results obtained in cellular studies, we decided to further examine compounds **1**–**5**. The effect of biguanides **1**–**5** on endothelial cell migration was investigated using an in vitro wound-healing assay. The test was performed using the JuLiStage device equipped with a camera which allows cell growth and migration to be visualized in real time. Metformin at 0.3 and 1.0 mM was used as a reference compound. HUVECs were monitored every 30 min over 24-h incubation. For the purposes of this publication, we analyzed the results at the starting point (T_0_), and after 6, 12, 18, and 24 h. The potential for metformin derivatives **1**–**5** to affect HUVEC migration is presented in Table S4 (Supplementary Materials). The results are expressed as the width of the scratched wound calculated using JuLiStage software.

**Figure 5:**
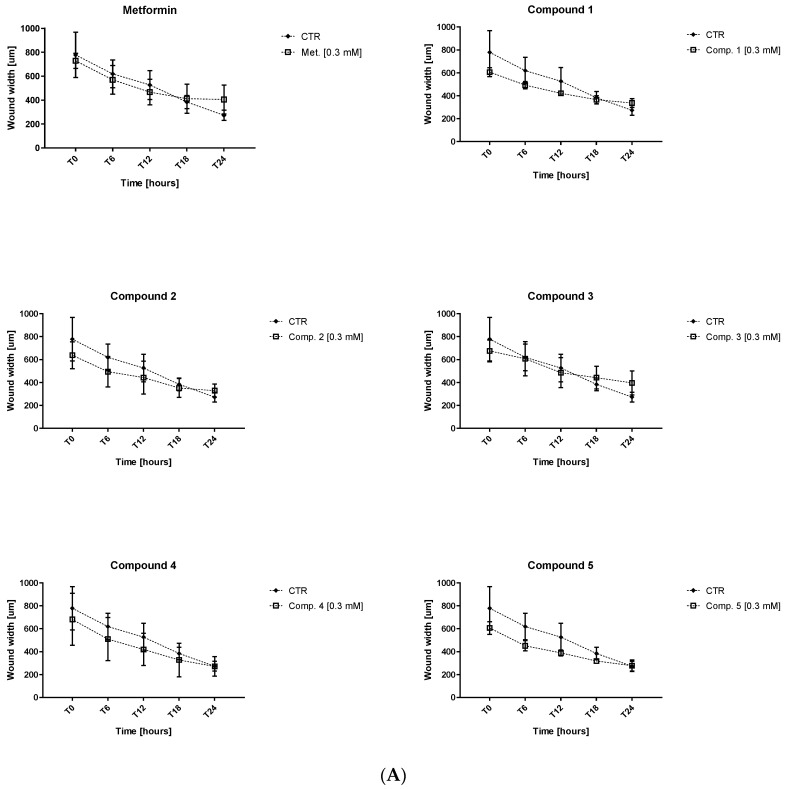

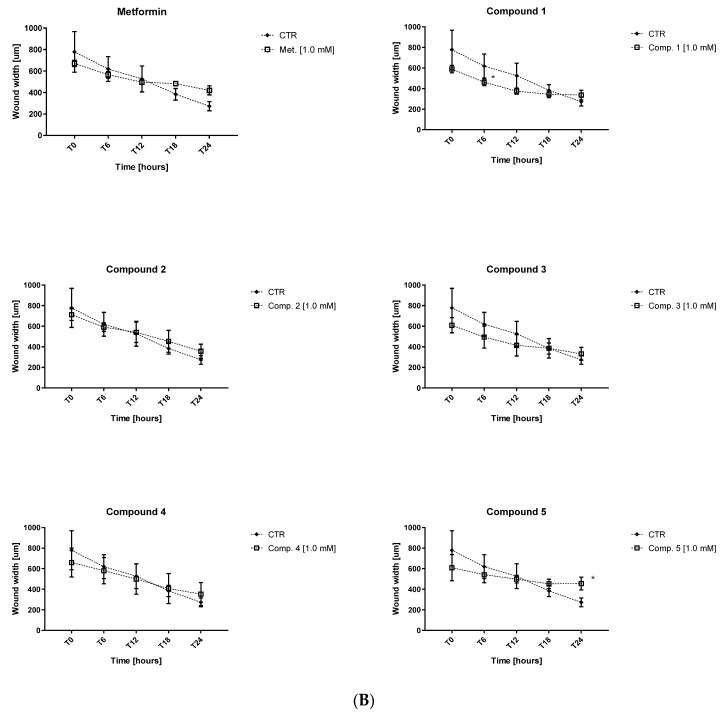
Impact of selected sulfonamides **1**–**5** and metformin on the migration of HUVECs was analyzed using wound-healing assay in the JulieStage system. The represented graphs reflect changes of the wound width (µm) during 24 h in the absence (control) and in the presence of selected compounds at the concentration of 0.3 (**A**) and 1.0 mM (**B**). The results are presented as mean ± SD, *n* = 4–8; (* *p* < 0.05).

Metformin was found to slightly modulate cell migration, which was manifested by an increase in the width of the wound in comparison with control (Figure 5, Table S4). For instance, the wound width was 419.8 ± 42.3 μm after 24 h of treatment with metformin (1.0 mM) and 272.4 ± 42.2 μm for controls. However, none of the changes reported for metformin were found to be statistically significant. Comparable results were also obtained for other tested compounds (**1**–**5**). The results of the conducted studies indicate that the tested compounds have the greatest influence on the migration of HUVEC after 24 h of incubation. However, statistically significant inhibition of cell migration was reported only in the case of compound **5** at 1.0 mM (*p* < 0.05). Although other compounds also demonstrated inhibition of HUVEC migration, these effects were not statistically significant.

These results were also visualized by a series of images of cells at various time points. Figure S3 (Supplementary Materials) presents representative images of wound closure in control cells and cells treated with metformin, compound **1**, and compound **5** at 1.0 mM. As depicted in Figure S3, metformin did not induce significant changes in the morphology and migration of HUVECs. Treatment of HUVEC with compound **1** and **5** resulted in greater numbers of round cells. Careful analysis of the images suggests that compound **5** moderately inhibited HUVEC migration after 24-h treatment.

### 2.5. Tissue Plasminogen Activator (t-PA) Release from Human Umbilical Endothelial Cells

The effects of compounds **1**–**5** on t-PA release from HUVEC were also tested at concentrations of 0.3 and 1.0 mM (Table 2). Metformin has previously been reported to significantly elevate t-PA release [28]; however, all tested compounds reduced the amount of t-PA released from the cells in a concentration-dependent manner. For instance, compound **1** significantly decreased t-PA concentration in cell supernatants to 2427.2 ± 246.0 pg/mL at 0.3 mM (*p* < 0.01) and 1709.5 ± 219.1 pg/mL at 1.0 mM (*p* < 0.001), compared with 2895.4 ± 205.7 pg/mL for control. The other compounds exhibited analogous properties towards t-PA release in HUVEC cell model.

### 2.6. Basic Coagulation Studies

The effects of the synthesized sulfonamides **1**–**5** on basic coagulation parameters are shown in Table 3. Compounds **6**–**8** were also tested for their impact on the intrinsic and extrinsic coagulation pathways; however, the cyano derivatives showed anti-coagulant activity only at the concentrations which are higher than their IC_50_ values toward HUVECs. The effects of biguanides on the activation of extrinsic coagulation pathway were evaluated using the Prothrombin Time (PT) test. Importantly, compound **1** prolonged Partially Activated Thromboplastin Time (APTT) (Table 3) at concentrations from 0.06 to 1.5 mM; however, the changes were not statistically significant at the highest concentration tested. In addition, derivative **5** significantly extended APTT at the concentration 1.5 mM, outside the reference values. However, it should be noted that these results were recorded at a concentration that inhibits the viability of HUVEC by 50%.

The tested compounds had varied effects on fibrin polymerization. For instance, compound **1** with alkyl chain significantly shortened Thrombin Time (TT) over the entire concentration range, while compound **2** with *n*-octyl chain affected TT at concentrations of 0.006–0.3 mM. In addition, compound **4**, with a trifluoromethyl substituent, and **5**, possessing an acetyl group, significantly extended TT outside the reference value range (14.0–18.0 s). For instance, compound **5** at the concentration 1.5 mM prolonged TT to 28.08 ± 1.49 vs. 16.30 ± 1.72 s for control. Similarly to the measurements of APTT, these results are reported for high concentrations being responsible for substantial inhibition of HUVEC growth.

### 2.7. Factor X Activity

Factor X is a vitamin K-dependent serine protease, which is synthesized in the liver and participates in both intrinsic and extrinsic coagulation pathways. Factor X deficiency can be inherited or brought on by severe liver disease, vitamin K deficiency, or the administration of anti-coagulant drugs such as warfarin; it can also occur in association with a variety of medical conditions, such as atypical chronic lymphoid leukemia. Factor X deficiency may contribute to the elongation of PT and APTT [33].

**Figure 6:**
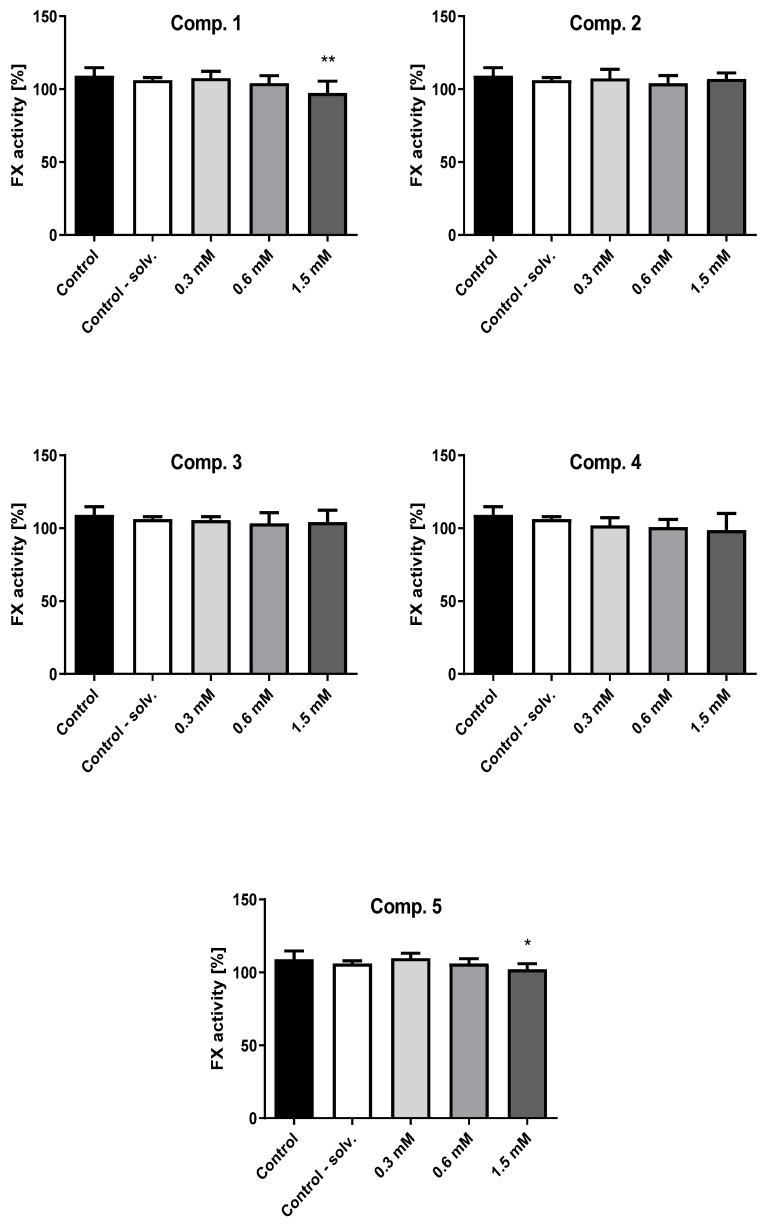
Effects of biguanides **1**–**5** on the activity of factor X (data are presented as mean ± SD; *n* = 4–5) after 1-min incubation with plasma-deficient factor X. The asterisk denotes a statistically significant difference between the samples treated with compounds and control; * *p* < 0.05, ** *p* < 0.01.

The impact of tested biguanides on the activity of factor X (%) are presented in Figure 6 and Table S5 (Supplementary Materials). Significantly lower factor X activity was observed for compounds **1**, and **5**, and this effect was concentration dependent. Compound **1** and **5** at the highest concentration significantly decreased the activity of factor X (*p* < 0.01, *p* < 0.05), which may be responsible for prolonged APTT (Table 3). However, in the case of compound **5**, it should be noted that these results were recorded for a concentration that inhibits the viability of HUVEC by 50%. The other compounds (**2**, **3**) did not exert any relevant influence on factor X activity.

### 2.8. Antithrombin III (AT) Activity

AT is a plasmatic α-glycoprotein, and is one of the most important plasmatic inhibitors of the activation of coagulation factors, such as thrombin, and free Xa, IXa, and VIIa plasmatic factors [34]. The activity of AT is measured by synthetic chromogenic substrate activity in the presence of excess heparin levels [35].

**Figure 7:**
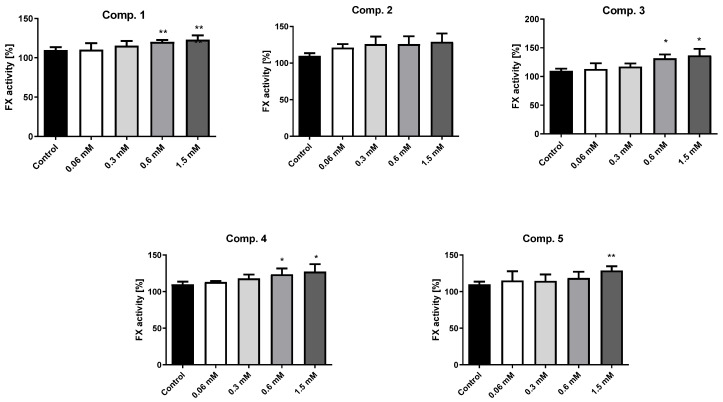
Effects of selected biguanides (**1**–**5**) on the activity of anti-thrombin III (AT) (data are presented as mean ± SD; *n* = 4). The asterisk denotes a statistically significant difference between the samples treated with compounds and control; * *p* < 0.05, ** *p* < 0.01.

Figure 7 depicts the effects of compounds (**1**–**5**) on the activity of AT (Table S6, Supplementary Materials). All studied compounds apart from compound **2** with *n*-octyl chain significantly increased the activity of AT. In the case of compound **1**, a significant elevation of AT activity was observed at 0.6 and 1.5 mM, and was 120.00 ± 2.71% and 123.00 ± 5.20%, respectively (*p* < 0.01). For instance, compound **3** also significantly increased the activity of AT to 131.75 ± 6.65 at 0.6 mM (*p* < 0.05) and 136.75 ± 11.32 at 1.5 mM (*p* < 0.05). However, the results reported for compounds **4** and **5** should not be regarded as important since they were recorded at the concentrations contributing to significant HUVEC death.

### 2.9. Clot Formation and Lysis Test (CL-Test)

**Figure 8:**
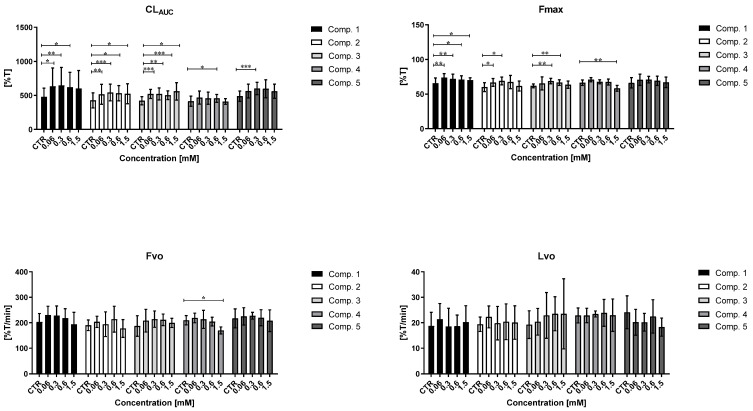
Influence of chosen biguanides **1**–**5** on overall potential of clot formation and fibrinolysis (CL_AUC_), and selected parameters of clot formation and fibrinolysis process: maximum clotting (Fmax); initial plasma clotting velocity (Fvo); initial clot fibrinolysis velocity (Lvo) (data are presented as means, *n* = 5–7). The asterisk denotes a statistically significant difference between the samples treated with compounds and respective controls; * *p* < 0.05, ** *p* < 0.01, *** *p* < 0.001. In our previous study, metformin did not affect the overall potential of clot formation and fibrinolysis (CL_AUC_) over the entire concentration range and dimethyl biguanide did not influence the kinetic parameters of the clot formation process and fibrinolysis (Fmax, Fvo, Lvo constants) [37].

Based on the results of the basic coagulation studies, compounds **1**–**5** were further tested for their impact on the overall potential of clot formation and fibrinolysis (CL_AUC_) using the CL-test [36]. This comprehensive analysis evaluates the effects of the examined sulfonamides on the kinetic parameters of the clot formation process, clot stabilization, and fibrinolysis by continuous monitoring of changes in transmittance (Figure 8, Table S7, Supplementary Materials).

#### 2.9.1. Overall Potential of Clot Formation and Fibrinolysis (CL_AUC_)

Overall, the compounds tested increased the overall potential of clot formation and fibrinolysis (↑ CL_AUC_), manifested by a statistically significant elevation of the area under the curve (AUC) of clot formation and fibrinolysis. Compound **4** stood out among the tested sulfonamides because it did not affect the overall potential of clot formation and fibrinolysis (CL_AUC_ constant) at the highest concentration (1.5 mM). These experiments also examined the overall duration of the clot formation and fibrinolysis process (T). Most of the tested compounds, apart from compound **5**, were found to significantly prolong the entire process at the highest concentrations (0.6–1.5 mM). However, similar to the study results discussed previously, these data were obtained at the highest concentrations that adversely affected the viability of HUVEC.

#### 2.9.2. Kinetic Parameters of Clot Formation Phase

One of the kinetic parameters of the clot formation phase is thrombin time (Tt), which expresses the time elapsed from the addition of exogenous thrombin (0.5 U/mL) to the beginning of clot formation. The obtained results showed that compounds **3** and **4** did not influence thrombin time (Tt constant), while compound **2** statistically significantly increased Tt at 1.5 mM (↑ Tt, *p* < 0.05). A detailed analysis of the data indicated that the tested compounds tended to shorten Tt when applied in the lower concentration range. Importantly, none of the compounds significantly affected the initial velocity of plasma clotting (Fvo constant), apart from compound **4** at 1.5 mM, which significantly decreased Fvo (↓ Fvo; *p* < 0.05). This change was reflected by a significant increase in plasma clotting time (↑ Tf) (*p* < 0.01). Incubation of human plasma with sulfonamide **4** at the highest concentration was also associated with a significant reduction of maximum clotting (↓ F_max_), which can be connected to its influence on the structure of the formed clot or adverse effect on the whole process of clot formation similarly to HUVEC viability. In contrast, compounds **1**, **2**, and **3** profoundly increased the maximum clotting (↑ F_max_), depending on the concentration.

#### 2.9.3. Kinetic Parameters of Clot Stabilization Phase

The second phase of the investigated process evaluated the clot stabilization time (Tc) and the area under the stabilization curve (Sc), as presented in Table S7 (Supplementary Materials). All compounds (**1**–**5**) significantly prolonged clot stabilization time (↑ Tc) and this effect was concentration dependent. These results indicated a delay in fibrinolysis. In addition, this alteration contributed to an increase in Sc, which suggests that these compounds may influence the clot structure.

#### 2.9.4. The Kinetic Parameters of the Fibrinolysis Phase

A statistically significant increase in maximum fibrinolysis (↑ Lmax) was observed for compound **2** (0.06–0.3 mM) (*p* < 0.01, 0.05, respectively), and this was related to the increase of maximum clotting value (↑ Fmax). These results indicate complete lysis of previously-formed clots. Importantly, none of the tested sulfonamides significantly affected the initial velocity of fibrinolysis (Lvo constant) over the entire concentration range, which suggests that the previously formed clots do not persist longer than in the case of control samples. The significant effect on the fibrinolysis time (↑ Tl) was only observed for compound **1** (0.3–0.6 mM) (*p* < 0.05), and it was related with the elevated Fmax. It should be highlighted that most of the studied compounds, particularly at a higher concentration range, did not contribute to any significant increase in the area under the fibrinolysis curve (Sl constant).

### 2.10. Coagulation Assay

Coagulation assay is an effective method assessing the impact of new compounds on the process of coagulation after generation of endogenous thrombin. Our studies revealed that compounds **2** (1.5 mM), **4** (0.6 mM), and **5** (0.06–0.3 mM) induced statistically significant changes in the length of thrombin generation time (↑ TGt), whereas compounds **1** and **3** did not affect TGt (Table S8, Supplementary Materials). A reduction in maximum clotting (Fmax) was observed for compounds **2** and **5** at the highest concentration (1.5 mM), and these data suggest that the chemical structure of these sulfonamides affected the clot structure. No such significant effect was observed for the other compounds (**1**, **3**, **4**), suggesting that they did not affect the clot structure after generation of endogenous thrombin. Importantly, compounds **4** at 0.3–0.6 mM and **5** at 0.3–1.5 mM significantly increased plasma clotting time (↑ Tf) and decreased initial plasma clotting velocity (↓ Fvo). In contrast, compounds **2** and **3** did not induce any significant influence on Fvo or Tf. Compounds **2** and **5** also decreased overall coagulation potential (↓S) at the highest concentration (*p* < 0.05), while coagulation potential remained constant for the others (S constant).

## 3. Discussion

Thanks to improvements in healthcare and nutrition, human life expectancy has greatly increased over the past few decades. However, an aging population is at greater risk of developing age-related diseases, including T2DM, cardiovascular diseases (CVD), neurodegenerative diseases (ND), and cancer. Despite our growing knowledge of their molecular basis, these conditions still lack effective novel treatments [38].

Of these conditions, the present paper places a strong emphasis on diabetes, which is a chronic, metabolic disease characterized by elevated blood glucose levels (hyperglycemia) and an impaired physiological balance between the coagulation and fibrinolysis processes described as diabetic thrombophilia; this is manifested by enhanced platelet aggregation capacity, increased coagulation factor activity, and hypofibrinolysis [39,40]. Microvascular complications in diabetes lead to an elevated risk of the occurrence of cardiovascular events, which are recognized as the main cause of the mortality associated with T2DM. Metabolic abnormalities related to diabetes such as hyperglycemia, hyperlipidaemia, and insulin resistance, as well as interrelated conditions including oxidative stress, endothelial dysfunction, and inflammation elevate the risk of platelet hyperreactivity and the development of hypercoagulabilty. Undoubtedly, hyperglycemia also has a negative influence on the vascular endothelium, which constitutes the primary defence against thrombosis. Endothelial impairment leads to enhanced permeability, vasodilation of blood vessels, and activation of clotting factors [41,42,43].

Nowadays, because of its glucose-lowering effects and good tolerance, weight-lowering properties, and low risk of hypoglycemia, metformin is regarded as a gold standard for the treatment of T2DM [39,44,45]. Although metformin exerts beneficial pharmacodynamic properties, it also possesses unfavorable pharmacokinetics properties, such as relatively slow incomplete absorption, moderate bioavailability, and significant intra-subject and inter-subject variability, which may result in the lack or poor response to the drug. Metformin itself is a strong base, which is administered orally in the form of a salt-N, N-dimethylbiguanide hydrochloride. Due to its strongly polar guanidine structure, metformin also exists as a highly hydrophilic cationic molecule (logP octanol: water = −2.6) under physiological conditions, which limits its passive diffusion through the cell membranes; this results in the drug only having 50%–60% bioavailability [23,31,46,47]. A crucial role in metformin transport across cell membranes and its accumulation in various organs is played by organic cation transporters (OCTs). These specific carriers are responsible for the oral absorption, distribution, elimination, and biochemical effects of metformin in the human organism [10,41,46,47].

A rapidly growing body of literature indicates that sulphonamides have a versatile structure and a broad range of bioactivity, which make them excellent candidates for drug repositioning [7]. Following on from our previous studies, and considering the favorable pleiotropic properties of metformin and the potential for sulfonamide application in medicines with multiple modes of action, the aim of the our present study was to evaluate a selection of eight new sulfonamide-based metformin analogues with glucose-lowering, anti-coagulant properties and effective intracellular uptake profiles as well as greater affinity towards Organic Cation Transporters (OCTs) [48], and to compare them with the parent drug.

Metformin is a glucose-lowering, insulin-sensitizing agent with a multidirectional mechanism of action. A key effect of metformin treatment is its potential for gluconeogenesis suppression based on inhibition of mitochondrial glycerolphosphate dehydrogenase (mtGPD) in hepatocytes [49,50]. Our findings suggested that all tested sulfonamides have the capability to elevate glucose utilization in HUVECs; however, statistically significant changes were observed for compounds **1**–**5**. Of the tested compounds, the alkyl derivatives (**1**–**3**) deserve special attention because they demonstrated the most comparable effects to the parent drug, metformin. A fuller appraisal of the effect of the newly-synthesized compounds on glucose uptake requires further experiments using other cellular models, such as hepatocytes and pancreatic and skeletal muscle cells.

There are two essential components to blood vessels: endothelial cells (ECs) and vascular smooth muscle cells (VSMCs), which are responsible for correct vascular development and the maintenance of cardiovascular homeostasis. The endothelium not only acts as a physical barrier between the vessel wall and lumen, but also secretes a number of mediators that regulate platelet aggregation, coagulation, fibrinolysis, and vascular tone [23,46,51,52]. In contrast, VSMCs are responsible for maintaining vessel stability, including their normal shape and their proper motor function, i.e., vasoconstriction and vasodilatation, through active contraction and relaxation. VSMC dysfunction has a key influence on the genesis and development of atherosclerosis and hypertension [53,54,55]. There is also some evidence that inflammatory activation of the endothelium promotes hypertension development [23,46].

The next step in our studies was to evaluate the effects of sulfonamide derivatives **1**–**8** on the growth of HUVEC and AoSMC using cellular in vitro models. All tested analogues demonstrated a comparatively lower influence on the viability of AoSMC than HUVECs, as indicated by WST-1 assay. Of all tested compounds, metformin analogues with alkyl tails (**1**–**3**) demonstrated greater safety, indicated by both cell lines demonstrating the highest viability. While, compounds **4** and **5** revealed moderate effects on HUVECs viability at the concentration up to ca. 1.0–1.5 mM. In contrast, compounds **6**–**8**, with a cyano moiety in the benzene aromatic ring, showed more potent inhibitory effects on HUVEC and AoSMC growth, resulting in considerably lower viability. Therefore, cyanobenzenesulfonamides appeared to be the most toxic of all tested compounds, and were not considered for further anti-coagulation studies.

The viability of AoSMCs incubated with derivatives **1**–**5** (3.0 mM) decreased to 56%–76% depending on the tested compound. Taking into account the resulting cellular viability values, 3.0 mM may be regarded as a safe concentration for further studies of these compounds. Furthermore, phase contrast microscopy suggested that the tested compounds exerted a less profound influence on AoSMC morphology (increase in the number of round cells) than on HUVEC morphology (presence of round and shrunken cells). Only the samples treated with compounds **4**–**8** demonstrated elongated cell bodies.

Vascular endothelial cell integrity was evaluated using the Real-Time Cell Electric Impedance Sensing (RTCA-DP) system, which is based on changes in the biological status of the cells (nCl parameter) following treatment with compounds **1**–**8**. For this purpose, the RTCA-DP system was used to determine cell number, proliferation rate, size, and shape, and to estimate the quality of cells adhering to the wells [56]. In the current study, a more significant decrease in HUVEC integrity was observed for sulfonamides **1** to **8** at concentrations, i.e., 1.0 mM, higher than 0.3 mM. For compound **1**, no significant influence on nCl value was identified after 36-h incubation for either concentration; this suggests that this sulfonamide derivative, with an *n*-butyl chain, exerted considerably smaller effects on HUVEC integrity, and did not appear to affect the immediate cellular response or cell adhesion, as reflected in the WST-1 assay results. The cyanobenzenesulfonamides (**6**–**8**) demonstrated the most unfavorable influences on cellular viability during the 36-h incubation period; an immediate decrease in CI value was observed, which continued throughout the entire co-treatment. The RTCA-DP and WST-1 data, as well as the morphology analysis, after 36-h incubation suggest that the observed reduction of nCI value is associated with profound changes in cellular viability. Therefore, compounds **6**–**8**, i.e., with a cyano group in the aromatic ring, were not examined in further studies due to their greater toxicity. It should be emphasized that the decreased cell adhesion and integrity observed following treatment with all other sulfonamide analogues do not appear to be associated with considerable alterations in cell viability.

External factors such as hyperglycemia, hyperlipidemia, hypertension, obesity, and inflammatory agents can induce endothelium activation, resulting in its dysfunction, excessive proliferation, and the formation of atherosclerotic plaques. As such, the modulation of EC metabolism may be a critical step in controlling the initiation and progression of atherosclerosis [57,58,59]. In addition to the loss of the endothelial barrier, atherosclerotic lesion development is also influenced by the migration and proliferation of smooth muscle cells (SMC) [60,61]. Therefore, compounds with potential inhibitory effects on the migration of pathological cells may be of value in suppressing atherosclerosis development.

The compounds were therefore subjected to endothelial cell migration study using the wound-healing assay. This was performed using the JuLiStage system, which allows real-time monitoring of live cells. The results indicated that metformin and their novel analogues (**1**–**5**) inconsiderably modulated cell migration during 24-h incubation. Only compound **5**, the acetylbenzenesulfonamide derivative, demonstrated some significant effects, i.e., a moderate suppression effect on endothelial cell migration. However, it should be noted that these effects might stem from inhibitory properties on HUVEC cell growth. The greatest inhibitory influence on HUVEC migration was achieved after 24-h stimulation.

Tissue plasminogen activator is a serine protease, which is synthesized in ECs occurring in blood plasma and is recognized as a marker of endogenous thrombolysis potential. The primary function of t-Pa includes catalyzing the conversion of plasminogen to plasmin, which is the primary enzyme involved in dissolving blood clots [62,63,64]. Metformin has previously been reported to significantly elevate t-PA release [28]; while, all tested compounds reduced the amount of t-PA released from the cells in a concentration-dependent manner. However, a review of the current academic literature does not provide an unequivocal answer in regard to the effect of metformin on the level of t-PA in both diabetic patients and healthy individuals, since the results of the studies indicated diversified effects. To deeply analyze the connection between the influence of the tested derivatives and metformin on the level of t-Pa and the ability to convert inactive plasminogen into active plasmin, it is of vital importance to conduct further in-depth studies to assess the activity of plasmin, the main enzyme converting fibrin into fibrin degradation products.

The hemocompatibility of newly-synthesized compounds is usually determined based on blood coagulation tests. In the present study, the quality of the extrinsic and intrinsic coagulation pathways was determined based on basic coagulation parameters including PT and APTT. Importantly, xenobiotics with shorter APTT may result in pro-coagulant imbalances, which would be associated with an enhanced risk of venous thromboembolism; in contrast, prolonged APTTs may indicate potential anti-coagulant properties [65,66,67].

The thrombin time is a clot-based test which measures the time required for fibrin clot formation following the addition of a standard amount of thrombin to plasma bypassing other coagulation factors. The results imply that compounds **4**, with trifluoromethyl substituent and **5**, with an acetyl group significantly prolonged TT. Not only did biguanide **5** affect the process of fibrin polymerization, reflected in longer TT, but it also inhibited the intrinsic coagulation pathway, indicated by APTT being prolonged to outside reference values. These beneficial effects were revealed by compounds **4**, **5** at concentrations of 0.6–1.5 mM, which are close to their IC_50_ values towards HUVEC viability (Table 1). However, the development of compounds that are able to improve basic hemostasis parameters at the beginning of preclinical studies is highly desirable, and may allow the development of new derivatives with more favorable anti-coagulant properties compared with the parent drug.

The extrinsic and intrinsic clotting pathways converge into a common path, of which the first element is factor X (FX), which converts prothrombin into thrombin. As such, our present studies examined the influence of compounds **1**–**5** on the activity of clotting factor X; the results provided an insight into their potential anti-coagulant properties. In particular, biguanide **1** significantly decreased the activity of the Stuart-Prower factor, which demonstrates that this derivative significantly influences APTT. Therefore, it appears that the new biguanide **1** analogue has more noticeable anti-coagulant activity than metformin.

It should be mentioned that in all types of diabetes, the main pro-coagulation agents are hyperglycemia and oxidative stress. Oxidative stress results in the inactivation of antithrombin III, overproduction of kallikrein, activation of the kininogenesis cascade, and excessive release of the antifibrinolytic factor PAI-1 from damaged endothelial cells in hyperglycemic states [62,68,69,70]. Therefore, the present study also estimated the action of biguanides **1**–**5** on the activity of AT. The results indicated that compounds **1** and **3**–**5** significantly increased AT activity, which can also explain their effects on measured APTT. These sulfonamides, and their capacity to significantly increase AT activity, confirmed highly beneficial anti-coagulant properties. It should be taken into account that significant elevation of AT for derivatives **4**–**5** was noted at concentrations that unfavorably affect the viability of HUVEC, resulting in its decrease of 50% (Table 1).

Compounds **1**–**5** were selected for further in-depth coagulation studies using the CL-test. This multiparametric method provides an insight into the effects of these compounds on the kinetic parameters of clot formation and fibrinolysis. Regarding the clot formation phase, the biguanide analogue with the *n*-octyl tail (**2**) significantly increased thrombin time (↑Tt), as did the trifluoromethyl derivative (**4**), which slightly prolonged Tt at the highest concentration; this was in accordance with the outcomes of the TT experiment. These data suggest that compounds **2** and **4** influence the process of fibrin polymerization. Similarly, compound **4** also significantly reduced initial clot formation velocity Fvo (↓Fvo; *p* < 0.05) and maximum clotting (↓Fmax) (*p* < 0.01) while increasing plasma clotting time (↑Tf) (*p* < 0.01). These findings suggest that compound **4** might have the capability to delay the process of clot formation by altering thrombin activity. It is worth noting that the concentrations at which compound **4** showed potential anti-coagulant properties were close or higher than its IC_50_ value towards HUVEC viability. Therefore, it can also be conjectured that this beneficial action of compound **4** can be also connected to its unfavorable effect on the whole process of clot formation, similarly to HUVEC viability, and it would require further in-depth studies. It should be also stressed that the remaining compounds did not significantly affect the initial velocity of plasma clotting (Fvo constant).

However, as both the initial velocity of fibrinolysis (Lvo) and fibrinolysis time (Tl) remained constant for compounds **2**–**5** over the entire concentration range, it appears that previously-formed clots did not persist longer than in the case of control samples; as such, they are not associated with any risk of slowing fibrinolysis. The obtained values for maximum lysis (Lmax) reflect those observed for Fmax, which suggests that previously-formed clots were completely lysed. However, most of the tested biguanides appeared to increase the overall potential of clot formation and fibrinolysis (↑CL_AUC_) with a concomitant increase of the overall duration of the entire process (↑T). The most neutral compounds towards the overall potential of clot formation and fibrinolysis, and kinetic parameters of this process, appeared to possess compounds **4** and **5**; however, the reported changes occurred only at the high concentrations contributing to the substantial decrease in the HUVEC viability (Table 1).

The overall potential of plasma coagulation is determined by a comprehensive appraisal of the coagulation process. In this method, the small amounts of thrombin and calcium chloride are used to induce a feedback reaction, which causes the generation of endogenous thrombin and subsequent coagulation [71]. During the experiments, we noticed that tested compounds **2** (1.5 mM), **4** (0.6 mM), and **5** (0.06–0.3 mM) significantly delayed the process of thrombin generation, and that this process was concentration dependent. Furthermore, sulfonamides **4** and **5** prolonged plasma clotting time (↑Tf) while decreasing the initial plasma clotting velocity (↓Fvo); this indicates that their chemical structure may influence the activity of thrombin. It is highly important to highlight that the reported changes occur at concentrations which are lower than compounds’ effects on HUVEC viability (Table 1). These results imply that the above-mentioned compounds can exert favorable effects on plasma coagulation. The only exception was compound **3**, which did not affect any of the coagulation parameters.

## 4. Materials and Methods

### 4.1. Materials

Basic coagulation studies were performed using Bio-Ksel reagents (Grudziądz, Poland): APTT reagent, calcium chloride, Bio-Ksel PT plus reagent (tromboplastin and solvent), and thrombin (3.0 UNIH/mL) for TT experiments. Calibrator (Bio-Ksel, Grudziądz, Poland), normal plasma (Bio-Ksel, Grudziądz, Poland), and water for injection (Polpharma, Gdańsk, Poland) were used for calibration and establishment of the coefficient of variation (for PT, APTT, TT studies).

Factor X activity was assessed with using factor X-depleted plasma (Bio-Ksel, Grudziądz, Poland), thromboplastin (Bio-Ksel, Grudziądz, Poland), and 0.9% saline. Tris buffer (50 mM) with heparin (2 U/mL) (Bio-Ksel, Grudziądz, Poland), chromogenic substrate (Bio-Ksel, Grudziądz, Poland) were used for antithrombin III measurements.

The CL-test used thrombin (Biomed Lublin, Lublin, Poland), recombinant tissue plasminogen activator (t-PA; Boehringer-Ingelheim (Ingelheim am Rhein, Germany), Tris-buffered saline (TBS; Polish Chemical Reagents, Gliwice, Poland), sodium chloride, and calcium chloride (Polish Chemical Reagents, Gliwice, Poland).

Human umbilical vein endothelial cells (HUVEC) were purchased from Lonza (Clonetics, Basel, Switzerland), and cultured according to the manufacturer’s guidelines. The following reagents were used for HUVEC maintenance: EGM-2—medium + bullet kit (Lonza, Clonetics, Basel, Switzerland), accutase (Sigma, St. Louis, MO, USA), and HEPES buffered saline solution (Lonza, Clonetics, Basel, Switzerland).

Human aortal smooth muscle cells (AoSMC) were purchased from ScienCell Research Laboratories (Carlsbad, CA, USA) and cultured according to the manufacturer’s guidelines. The AoSMC cell culture medium included 500 mL basal medium (SMC, ScienCell Research Laboratories, Carlsbad, CA, USA), 10 mL fetal bovine serum (FBS, ScienCell Research Laboratories, Carlsbad, CA, USA), 5 mL smooth muscle cell growth supplement (SMCGS, ScienCell Research Laboratories, USA), and 5 mL penicillin/streptomycin solution (ScienCell Research Laboratories, Carlsbad, CA, USA). The AoSMCs were passaged in trypsin/EDTA (ScienCell Research Laboratories, Carlsbad, CA, USA), Trypsin Neutralizing Solution (ScienCell Research Laboratories, Carlsbad, CA, USA). Poly-L-Lysine (ScienCell Research Laboratories, Carlsbad, CA, USA) was used to coat the culture flasks.

Glucose uptake studies were conducted using 2-NBDG (2-(N-(7-Nitrobenz-2-oxa-1,3-diazol-4-yl)Amino)-2-Deoxyglucose (Thermo Fisher Scientific, Invitrogen, Waltham, MA, USA). Cell viability was estimated using WST-1 assay (Takara, Takara Bio Europe, Saint-Germain-en-Laye, France). E-Plate 16 View (Roche & ACEA Biosciences, Santa Clara, CA, USA) and phosphate-buffered saline (PBS, Biomed Lublin, Lublin, Poland) were used for estimation of integrity of HUVECs in the real-time cell electric impedance sensing system. Human tissue plasminogen activator ELISA kit (Abcam, Cambridge, UK) was used for evaluation of t-PA concentration in the HUVEC supernatants.

### 4.2. Studied Compounds

The synthesis protocol, chemical characterization, and basic properties of the studied compounds **1**–**8** (Figure 1) were described previously [48]. All compounds were found to be stable in Tris buffer and human plasma [48].

### 4.3. Plasma Preparation for Basic Coagulology Tests

The experiments on the biological material were accepted by the Bioethics Committee of the Medical University of Lodz (Medical University of Lodz, Poland, approval no. RNN/105/20/KE). The studies on human biological material were conducted in accordance with Polish national directives. The tested human blood constituted a residual material of routine diagnostic studies destined for removal as medical waste.

The blood was collected into vacuum tubes stuffed with 3.2% buffered sodium citrate. Platelet poor plasma (PPP) was obtained by centrifugation at 3000× *g* for 10 min at room temperature using a Micro 22R centrifuge (Hettich Zentrifugen, Tuttlingen, Germany). The obtained PPP was stored in small aliquots for up to one month at –30° C. Before each experiment, PPP was restored at 37 °C for 15 min. Once thawed, the PPP was not frozen again nor used for retesting.

### 4.4. Glucose Utilization Assay

HUVEC were seeded at a density of 20,000 cells per well in 48-well plates, and were cultured in standard conditions for 24 h (medium EGM-2; volume 0.2 mL). Following this, compounds **1**–**8** were added at concentrations of 0.1 and 0.3 mM, and the HUVECs were incubated for another 24 h. Cells treated with ᴅ-glucose (final concentration in a sample 0.1 mM) were used as positive controls. On the following day, the cells were rinsed with PBS (Biomed Lublin, Lublin, Poland) and cultured for two hours in glucose-free DMEM medium supplemented with 1% BSA (bovine serum albumin, Sigma, St. Louis, MO, USA). Then, the cells were co-treated with insulin (final concentration 0.0001 mM) for 30 min. Afterwards, the medium was discarded and the cells were treated with 2-NBDG (final concentration 0.05 mM) for 30 min, followed by removal of the solutions of each well. The cells were washed twice with 100 µL PBS, and lysed using 1% Triton X-100 solution in PBS. The fluorescence was recorded using a microplate reader (Biotek, Instruments, Winooski, VT, USA) at 480/530 nm.

The concentration of intracellular 2-NBDG was calculated using a calibration curve obtained using 2-NBDG at concentrations ranging from 0.0001 to 0.025 mM in lysis buffer. The results are presented as mean ± SD, *n* = 4–6. The coefficient of variation for the assay was calculated (CV = 2.2%, *n* = 8).

### 4.5. Cell Viability Assay

HUVEC and AoSMC viability were tested using the WST-1 cell viability assay (Takara Bio Europe, Saint-Germain-en-Laye, France). The study protocol is given in detail elsewhere [72]. Briefly, HUVEC and AoSMC were seeded at densities of 7500 and 5000 cells per well on 96-well plates. The cells were cultivated for up to 24 h to achieve 70% confluency, and subsequently treated with tested compounds diluted in medium (1 + 9; v = 100 μL) or pure medium (control). The plates were incubated at 37 °C (5% CO_2_) for the next 24 h. Then, the cells were rinsed with 100 μL culture medium, and WST-1 reagent diluted in medium (100 μL) was added; the plates were then incubated again (37 °C, 5% CO_2_) for another 90 min. The absorbance was measured at 450 nm (iMARK, Bio-Rad, Hercules, CA, USA). The experiments were performed in multiplicate (*n* = 8) and the results were presented as mean ± SD. The cell viability was expressed as a percentage of the control samples, which constituted 100% viability. Where possible, IC_50_ values (the concentration of tested compound inhibiting cell growth by 50%) were calculated using concentration-response curves (GraphPad Prism 5, San Diego, CA, USA). The variability coefficient of the method was estimated (CV_(HUVEC)_ = 8.94%, *n* = 8; CV_(AoSMC)_ = 5.84%, *n* = 10).

The impact of the tested derivatives on HUVEC and AoSMC morphology was examined using an inverted microscope with phase contrast (magnification 100×) (Opta-Tech, Warszawa, Poland, software OptaView 7).

### 4.6. HUVEC Integrity Studies

The effects of sulfonamides **1**–**8** on the integrity and barrier properties of HUVEC were examined in real-time using a cell electric impedance monitoring system (Real-Time Cell Analyzer; Roche & ACEA Biosciences, Santa Clara, CA, USA), which is based on tracking electrical impedance signals. Any changes in cell status, adhesion, and morphology are expressed by parameter Cell Index (CI) [73]. The experimental protocol has been described previously [28]. Briefly, the cells were seeded at a density of 15,000 cells per well on E-16 plates (Roche & ACEA Biosciences, Santa Clara, CA, USA), and cultured until the cells achieved plateau phase. Then, the medium was discarded from each well, and the solutions of the test compounds dissolved in cell culturing medium (100 μL) or pure medium (100 μL, control) were added. The measurements were then carried out for 48 h. The results are presented as mean ± SD of ‘normalized cell index’ (nCI), which is calculated by the division of a CI value at a certain time point by the CI value at a reference time point. The experiments were conducted in multiplicate (*n* = 4–6). The method was validated and the coefficient of variability (CV) was calculated as 9.17%–15.2%, depending on the measured time point (*n* = 6).

### 4.7. HUVEC Migration in Real Time

The effects of compounds **1**–**5** on HUVEC migration were determined using the JuLI™ Stage system (NanoEntek, Seoul, Korea): a real-time cell history recorder allowing for live cell imaging and analysis [74]. The procedure for the migration experiments has been published elsewhere in more detail [74]. Concisely, HUVECs were seeded at a density of 10,000 cells per well on 96-well plates and incubated at standard conditions for 24 h. Following this, a wound was made with a scratcher (NanoEntek, Seoul, Korea). The cells were then rinsed with 100 μL of fresh EGM2 medium, and treated with biguanides **1**–**5** at the concentrations of 0.3 and 1.0 mM. The cells were incubated and monitored continuously every 10 min for 36 h at 37 °C, 5% CO_2_. Analysis of obtained images of cell migration, and measurements of the width of the scratch area were performed using dedicated software (NanoEntek, Seoul, Korea). The results were presented as mean ± SD, *n* = 4–8. The CV for the method was estimated as 14.2–24.4%, depending on the time point (*n* = 8).

### 4.8. Tissue Plasminogen Activators Release from Endothelial Cells

The influence on tissue plasminogen activator release from HUVECs was assessed using an ELISA kit (Abcam, Cambridge, UK). HUVECs seeded at a density of 10,000 per well on 96-well plates were co-incubated with compounds **1**–**5** at the concentrations of 0.3 and 1.0 mM for an additional 24 h (37 °C, 5% CO_2_). The cell supernatants were discarded into Eppendorf tubes and preserved at −20° C until analysis. Before the tests, the samples were thawed at room temperature for 15 min, and diluted 2.5-fold with diluent in Elisa kit. The measurements of t-PA concentrations in cell supernatants were conducted according to the manufacturer’s protocol using 3,3’,5,5’-tetramethylbenzidine (TMB) as a chromogenic substrate. The concentration of t-PA in HUVEC supernatants was determined using a calibration curve (R^2^ = 0.9936). The results are presented as mean ± SD, *n* = 6. The CV for the applied method was 7.21%.

### 4.9. Basic Coagulation Tests: PT, INR, APTT, TT

The effects of biguanidines (**1**–**5**) on the basic coagulation parameters (PT, INR, APTT, and TT) were carried out using a CoagChrom-3003 coagulometer (Bio-Ksel, Grudziądz, Poland), according to the routine laboratory diagnostic procedure [37]. The studies were performed in multiplicate (*n* = 4–7). The control samples containing distilled water and methanol (1:1) were also included.

The results are presented as mean ± standard deviation (SD). The methods were validated, and coefficients of variability for all experiments were calculated (PT: CV = 10.25%, APTT: CV = 8.55%, TT: CV = 4.87%). The reference values for each test are as follows: PT: 9.7–14.6 s; APTT: 26.7–40.0 s; TT: 14.0–18.0 s for 3.0 UNIH/mL of thrombin.

### 4.10. Factor X Activity

The activity of FX was measured optically using deficient plasma factor X [75]. The content of each sample was as follows: 50 µL of four-fold diluted PPP, 50 μL of deficient plasma factor X, 10 μL of the examined compound, and 100 µL PT reagent. The control samples contained 10 μL of distilled water and methanol (1:1). The measurement of PT time was recorded using a CoagChrom-3003 coagulometer (Bio-Ksel, Grudziadz, Poland). The activity of factor X was counted using a calibration curve (R^2^ = 0.999) performed on a calibrator diluted in the range 1:5–1:80 [76]. The variation in plasma coagulation time is proportional to the concentration and activity of factor X in the patient plasma treated with the tested compounds. The coefficient of variability for the method was calculated as CV = 1.80%. The reference values for factor X activity ranged from 77–131%.

### 4.11. Activity of Antithrombin III (AT)

The activity of AT in human plasma was estimated spectrophotometrically using a diagnostic laboratory method [75]. The test samples consisting of pre-warmed PPP diluted 30-fold (50 µL), thrombin (10 U/mL) dissolved in Tris buffer (50 mM) in the presence of excess heparin (2 U/mL) (50 µL), and 10 µL of examined compounds were incubated for one minute (37 °C). In the control samples, 10 μL of distilled water and methanol (1:1) was used. The reaction was initiated by the addition of a chromogenic substrate dissolved in water for injection; as a result, *p*-nitroaniline was released proportionally to the level of AT. The evaluation of AT level was performed on the basis of a calibration curve performed on calibrator diluted in the range 1:30–1:120 (λ = 405 nm) by the coagulometer program (CoagChrom-3003 Bio Ksel, Grudziadz, Poland). The coefficient of variability for the method (CV) was 2.69%, and the reference range was 80%–130%.

### 4.12. Clot Formation and Fibrinolysis Test (CL-Test)

The effects of the selected sulfonamide derivatives on overall hemostasis potential by means of clot formation and fibrinolysis test (CL-test) were further tested based on the continuous measurement of the changes in optical transmittance over time, as described by Kostka et al. [36] and Sikora et al. [71]. These experiments were conducted using 470 µL of three-fold diluted human citrate plasma, 10 µL of the tested compounds, and 10 µL t-PA (final concentration in a sample 220 ng/mL). The clot formation was triggered by thrombin (10 µL, final concentration 0.5 IU/mL). The clot formation and lysis curves were determined at a wavelength of 405 nm with a spectrophotometer (Cecil CE 2021; London, UK) with circulating thermostated water (37 °C) and a Model 300 Electronic Stirrer (Rank Brothers Ltd., Cambridge, UK). The experiments were conducted in multiplicate (*n* = 5–7), and the results are presented as mean ± SD.

The received graphs were analyzed using dedicated software [36] to determine the kinetic parameters of clot formation, stabilization, and fibrinolysis. The following parameters of clot formation were determined: Tt—thrombin time (s), Fmax—maximum clotting (%T), Tf—plasma clotting time (s), Fvo—initial plasma clotting velocity (%T/min), Sr—area under the clot formation curve (%Txmin). The parameters of the clot stabilization phase comprised Tc—clot stabilization time (s), Sc—area under the curve of stable clot formation (%Txmin). The parameters of fibrinolysis comprised Lmax—maximum lysis (%T), Tl—fibrinolysis time (s), Lvo—initial clot fibrinolysis velocity (%T/min), Sf—area under the fibrinolysis curve (%Txmin). Additionally, the overall potential of clot formation and fibrinolysis (CL_AUC_, (%Txmin)) and total time of the process of clot formation and fibrinolysis (T, (s)) were also estimated.

The method of clot formation and fibrinolysis was validated, and the coefficient of variation (CV) for pooled human plasma (*n* = 7) was within the range of 3.74–14.53% depending on the calculated parameter [74].

### 4.13. Coagulation Assay

The coagulation assay is a modification of the CL-test, in which a high concentration of thrombin (0.5 IU/mL) is replaced by small amounts of thrombin (0.00312 IU/mL) and calcium chloride (5 mM). These reagents induce a feedback reaction leading to the generation of endogenous thrombin, and coagulation [37,71]. The other experimental conditions were the same as for the CL-test (Section 4.12).

The obtained curves were analyzed using dedicated software [36] that estimates the following parameters: TGt—thrombin generation time (s), Fmax—maximum clotting (%T), Tf—plasma clotting time (s), Fvo—initial plasma clotting velocity (%T/min), Sf—area under the clot formation curve (%Txmin), Tc—clot stabilization time (s), S—area under the curve of coagulation (%Txmin).

### 4.14. Statistical Analysis

Statistical analysis was performed using Statistica 12.0 (StatSoft, Kraków, Poland) and GraphPad Prism 5 software (San Diego, CA, USA). The Shapiro-Wilk test was used to check the normality of the distribution of continuous variables, while the homogeneity of variances was verified using Levene’s test. The paired *t*-test was performed to test the dependent variables (e.g., studies on biological material), while statistically significant differences between the means of independent groups were identified using one-way ANOVA. The results of all the tests were considered significant at *p*-values lower than 0.05.

## 5. Conclusions

This paper described a biological evaluation of the effects of eight novel sulfonamide-based derivatives of metformin on vascular hemostasis, plasma properties, and cell function using experimental in vitro models. Our novel findings demonstrate that sulfonamides with alkyl chains (**1**–**3**), trifluoromethyl substituent (**4**), or an acetyl group (**5**) significantly increased glucose uptake in HUVECs, and that the obtained data are comparable to those received for the parent drug, metformin.

It should be emphasized that metformin analogues with alkyl tails (**1**–**3**) did not affect either HUVEC or AoSMC viability at concentrations up to 3.0 mM. In contrast with the above-mentioned compounds, derivatives **4** and **5** revealed a more toxic influence to HUVEC cells at the concentration of ca. 1.0–1.5 mM. These results are in agreement with those received in HUVEC integrity studies using RTCA DP. Studied compounds exerted comparable effects on HUVEC migration in wound healing assay to the parent drug, metformin.

Previous studies indicate that metformin affects neither the intrinsic or extrinsic coagulation pathway (PT, APTT), nor the kinetic parameters of clot formation or fibrinolysis (CL-test), nor the activity of factor X, nor HUVEC or AoSMC viability within the concentration range 0.006–3.0 mM [37,75,77]. Taken together, our research indicated that among all tested compounds, butylsulfonamide (**1**), octylsulfonamide (**2**), and cyclohexylsulfonamide (**3**) deserve special attention because they not only demonstrated the greatest safety profile on both cell lines but also significantly increased glucose uptake compared with the parent drug, metformin. Importantly, these compounds beneficially affect some parameters of plasma hemostasis, and therefore exert more profound anti-coagulation activity than metformin. In addition, our present findings suggest that trifluoromethanesulfonamide (**4**) and *p*-acetylbenzenesulfonamide (**5**) exhibit both anti-hyperglycemic properties and highly desirable anti-coagulant activity; however, these properties were reported at high concentrations, frequently similar to their IC_50_ values towards HUVECs, which give rise the safety issue of these two compounds. Although some of the obtained outcomes appear promising, further in vivo studies should be conducted to obtain a model of the complete effectiveness and safety of these new metformin analogues. To conclude, the chemical modification of the metformin backbone into sulfonamides with various substituents appears a promising point of departure for the development of novel metformin derivatives with stronger anti-coagulant properties.

## Figures and Tables

**Table 1:** The effects of sulfonamides **1**–**8** on HUVEC and AoSMC cells growth. The results (IC_50_ values, mM) are presented as mean ± SD (*n* = 6–8).

	Viability-IC_50_ [mM]	
**Compound**	HUVEC cells	AoSMC cells
**1**	3.643 ± 0.26	>59% at 3 mM
**2**	n.d.	3.710 ± 0.54
**3**	3.506 ± 0.24	>76% at 3 mM
**4**	1.038 ± 0.15	>56% at 3 mM
**5**	1.589 ± 0.13	>62% at 3 mM
**6**	0.461 ± 0.13	1.685 ± 0.21
**7**	0.304 ± 0.12	1.891 ± 0.16
**8**	0.791 ± 0.13	1.573 ± 0.14

n.d.—not determined; at 3.0 mM the viability was 41.5%, and at 5 mM viability was 32.1%.

**Table 2:** Effects of sulfonamides **1**–**5** on the release of t-PA from HUVEC cells. The results (t-PA concentration in supernatants, pg/mL) are presented as mean ± SD (*n* = 6). Values in bold are statistically significant in comparison with control samples. An asterisk denotes a significant difference between tested compounds and control samples (** *p* < 0.01; *** *p* < 0.001).

Compound	Concentration (mM)	Released t-PA (pg/mL)
**CTR**	-	2895.4 ± 208.7
**1**	0.3	2427.2 ± 246.0 **
	1.0	1709.5 ± 219.1 ***
**2**	0.3	1633.9 ± 263.2 ***
	1.0	990.0 ± 158.5 ***
**3**	0.3	1550.9 ± 135.2 ***
	1.0	1267.1 ± 350.6 ***
**4**	0.3	1958.3 ± 175.3 ***
	1.0	1481.8 ± 134.0 ***
**5**	0.3	1947.0 ± 207.3 ***
	1.0	1326.2 ± 149.4 ***

**Table 3:** The effects of compounds **1**–**5** on basic coagulation parameters.

Compound	Concentr. (mM)	PT (s)	INR	APTT (s)	TT (s)
**1**	0 (CTR)	12.46 ± 1.49	1.00 ± 0.12	29.36 ± 2.23	15.50 ± 0.92
	0.006	13.04 ± 2.41	1.04 ± 0.19	35.12 ± 4.75	14.03 ± 0.94 **
	0.06	12.78 ± 1.86	1.11 ± 0.23	33.36 ± 1.78 **	12.98 ± 1.08 **
	0.3	13.46 ± 2.71	1.08 ± 0.22	33.76 ± 2.53 *	13.90 ± 1.24 *
	0.6	13.08 ± 2.36	1.05 ± 0.19	34.68 ± 3.31 *	12.64 ± 1.31 **
	1.5	13.48 ± 2.93	1.08 ± 0.23	34.68 ± 5.21	13.03 ± 0.86 **
**2**	0 (CTR)	12.38 ± 1.68	0.99 ± 0.13	30.77 ± 4.03	15.76 ± 0.36
	0.006	13.30 ± 1.81	1.07 ± 0.15	30.79 ± 5.87	14.14 ± 0.59 ***
	0.06	12.82 ± 1.33	1.03 ± 0.10	31.33 ± 5.53	13.86 ± 0.80 **
	0.3	13.10 ± 1.37	1.05 ± 0.11	33.49 ± 4.85	13.48 ± 2.10 **
	0.6	12.96 ± 1.42	1.04 ± 0.11	33.46 ± 5.59	14.40 ± 2.35
	1.5	13.20 ± 1.23	1.06 ± 0.10	34.83 ± 6.22	16.32 ± 2.21
**3**	0 (CTR)	12.60 ± 2.54	1.01 ± 0.20	30.90 ± 2.92	15.58 ± 1.07
	0.006	12.52 ± 1.42	1.00 ± 0.11	32.97 ± 5.06	13.18 ± 1.97 *
	0.06	13.02 ± 1.90	1.04 ± 0.15	34.50 ± 4.52	13.14 ± 2.01 *
	0.3	13.60 ± 1.39	1.09 ± 0.11	33.00 ± 5.70	13.46 ± 1.97 **
	0.6	13.66 ± 2.27	1.09 ± 0.18	33.17 ± 5.34	13.50 ± 1.74 **
	1.5	13.30 ± 2.30	1.06 ± 0.18	32.49 ± 7.50	14.06 ± 1.60 **
**4**	0 (CTR)	13.26 ± 2.06	1.06 ± 0.14	37.30 ± 6.38	16.00 ± 0.78
	0.006	12.18 ± 2.09	1.00 ± 0.15	33.04 ± 4.10	16.55 ± 1.11
	0.06	11.80 ± 1.24	0.93 ± 0.10	35.22 ± 2.68	14.83 ± 1.11
	0.3	11.38 ± 1.26	0.90 ± 0.10	34.14 ± 3.41	17.88 ± 3.58
	0.6	11.80 ± 0.39	0.95 ± 0.03	34.40 ± 4.46	17.78 ± 2.40
	1.5	12.02 ± 0.79	0.96 ± 0.07	43.85 ± 6.20	22.03 ± 2.29 **
**5**	0 (CTR)	12.90 ± 1.80	1.06 ± 0.13	34.80 ± 3.08	16.30 ± 1.72
	0.006	13.70 ± 3.41	1.09 ± 0.27	35.06 ± 5.82	16.55 ± 1.57
	0.06	11.52 ± 0.86	0.92 ± 0.07	32.66 ± 3.72	16.70 ± 2.48
	0.3	11.88 ± 1.08	0.95 ± 0.09	32.38 ± 2.48	18.48 ± 1.34
	0.6	11.98 ± 0.97	0.96 ± 0.08	36.10 ± 4.40	23.30 ± 2.25 *
	1.5	13.44 ± 1.18	1.05 ± 0.09	77.52 ± 9.85 ***	28.08 ± 1.49 ***

The results are presented as mean ± standard deviation, *n* = 4–7. Values in bold are statistically significant in comparison with control samples. * *p* < 0.05, ** *p* < 0.01, *** *p* < 0.001. The reference values: PT: 9.7–14.6 s; INR: 0.85–1.15; APTT: 26.7–40.0 s; TT: 14.0–18.0 s for 3.0 UNIH/mL of thrombin.

## Data Availability

The datasets generated during the current study are available from the corresponding author on reasonable request.

## Supplementary Materials

The following are available online at https://www.mdpi.com/article/10.3390/ijms23084132/s1.
