# Supplementary material for: Structural Comparison of Sulfonamide-Based Derivatives That Can Improve Anti-Coagulation Properties of Metformin
Source: Int J Mol Sci. 2022 Apr 8;23(8):4132. doi: 10.3390/ijms23084132 (PMC9029893; doi:10.3390/ijms23084132)
Supplement: Supplementary file 1 [file ijms-23-04132-s001.zip › ijms-1626044-supplementary.pdf]

**Figure S1.** Effects of metformin and biguanide analogues **6** – **8** at the concentration of 0.1 (A) and 0.3 mM (B), and ᴅ-glucose at the concentration of 0.1 mM on the 2-NBDG uptake in HUVEC cells. The results are presented as mean ± SD, n = 4 – 6, an asterisk denotes the significant differences between control and cells treated with glucose, metformin or studied compounds. * $p < 0.05$; *** $p < 0.001$.

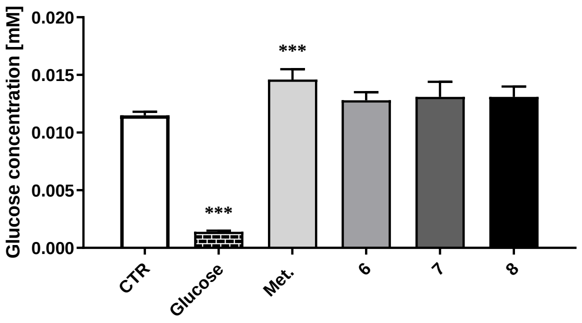


**(A)**

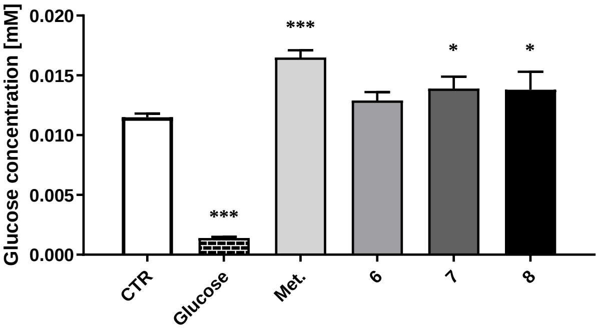


**(B)**

**Figure S2.** The effects of biguanides **6** – **8** on the integrity of HUVECs analyzed in the RTCA-DP system. A) The effect of the exposure of compounds **6** –**8** on normalized Cell Index (nCI) at selected time points (3, 6, 12, 24 and 36 hours). The results are presented as mean ± SD, n = 4 – 5. An asterisk denotes a significant difference between sample treated with biguanide and control sample; *** $p < 0.001$. B) The effects of compound **7** on the barrier properties of HUVECs. The picture presents representative plots of one experiment conducted in duplicates (the results are presented as a mean (solid line) ± standard deviation). For the statistical analysis there were conducted three independent experiments. Red line – control (unstimulated cells); green line – compounds at the concentration of 0.3 mM; navy blue line - 1.0 mM.

A)

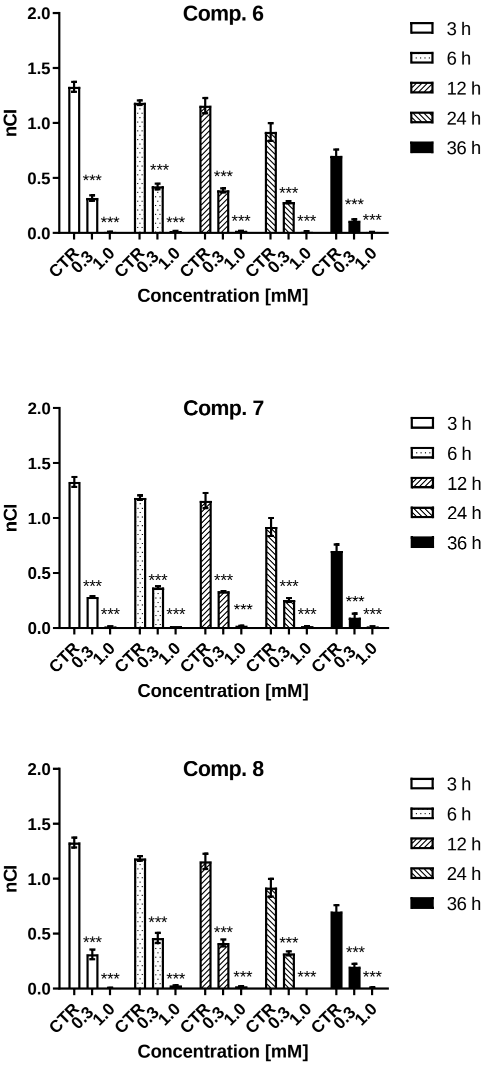

B)

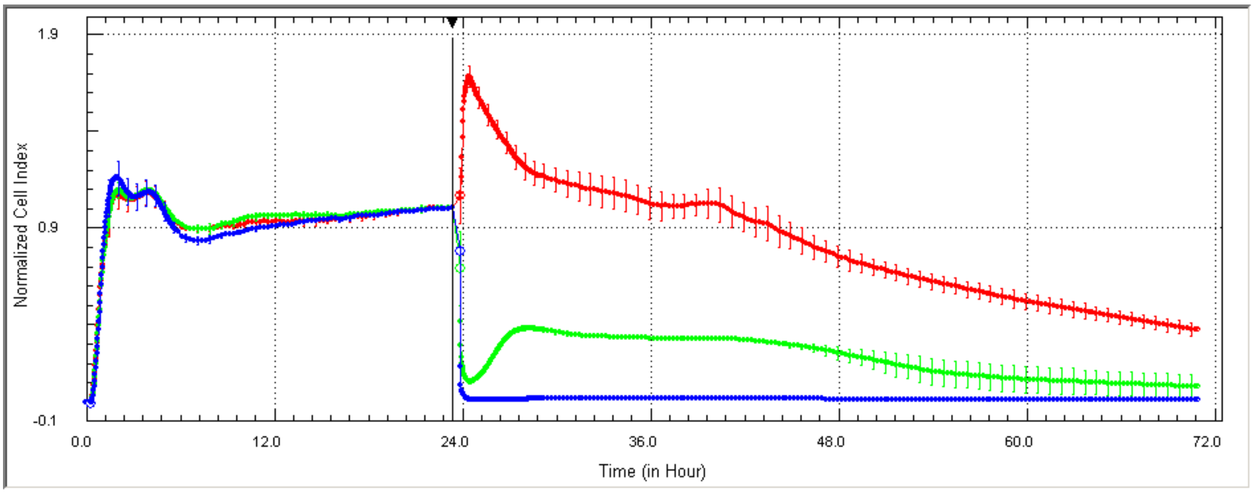


Metformin was not found to affect the integrity and adherence of HUVEC cells over the entire concentration range (0.006 – 1.5 mM) in our previous study [30]. For instance, after 24 hours of co-stimulation with metformin at 1.5 mM nCI was 0.95 ± 0.08 versus control 1.01 ± 0.07 [30].

**Figure S3.** HUVEC migration in the presence of metformin and selected biguanides (compound **1** and **5**). Cell migration was evaluated using wound healing assay in JuLiStage system. Representative cell images are shown for control samples (CTR), metformin, compound **1** and **5** at the concentration of 1.0 mM. Cells were photographed at the indicated time points (t = 0, 6, 12, 18, 24 h); 40× magnification.

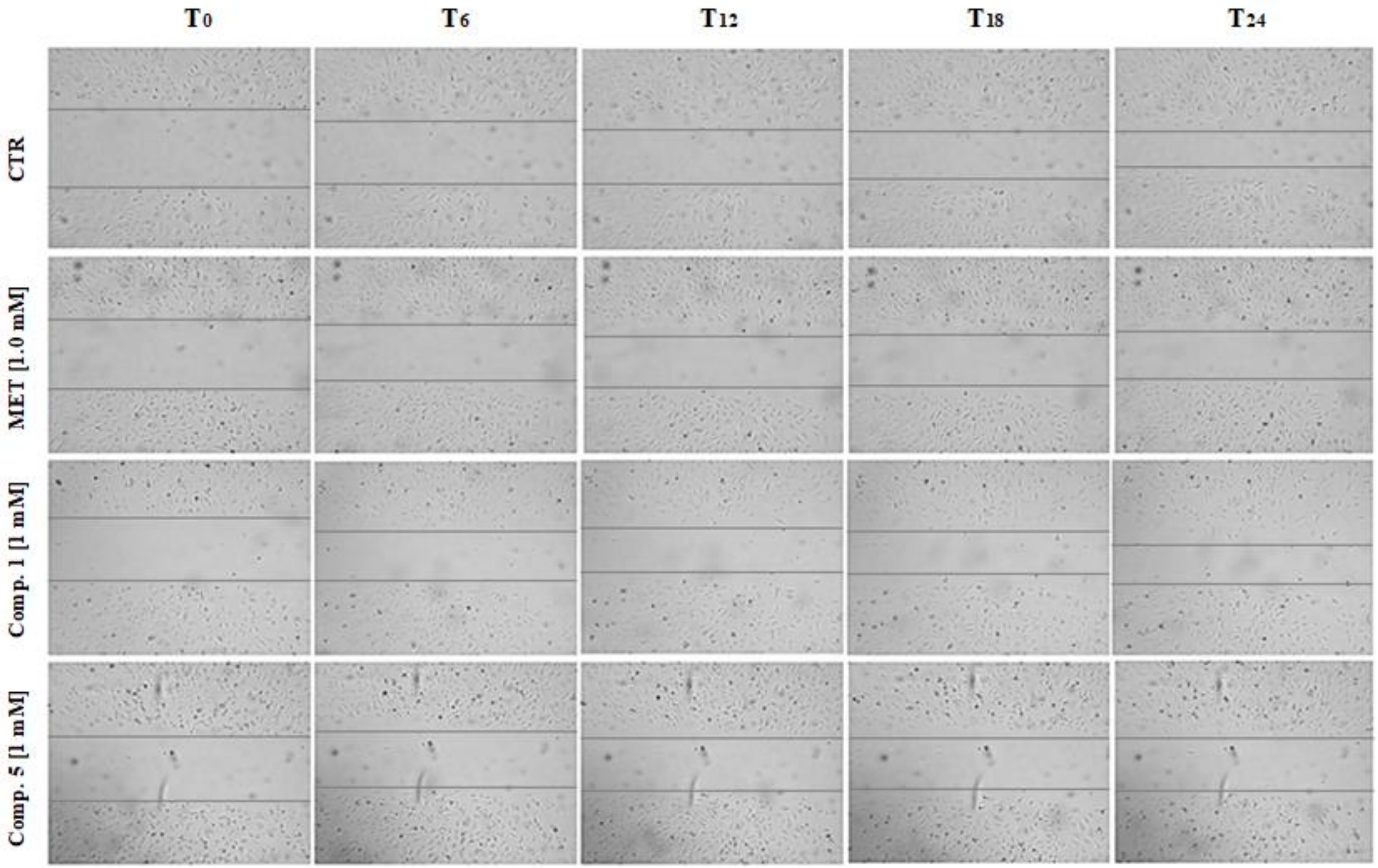

**Table S1.** Effects of biguanides **1 – 8** on the intracellular glucose uptake. The results are presented as mean ± SD, n = 4.

| **Compound** | **Concentration [mM]** | **Glucose uptake [mM]** |
|---|---|---|
| **Control** | - | 0.0115 ± 0.0003 |
| **Glucose** | 0.1 | **0.0014 ± 0.0001*** ** |
| **Metformin** | 0.1 | **0.0146 ± 0.0009*** ** |
| | 0.3 | **0.0165 ± 0.0006*** ** |
| **Comp. 1** | 0.1 | **0.0134 ± 0.0004** ** |
| | 0.3 | **0.0142 ± 0.0007*** ** |
| **Comp. 2** | 0.1 | **0.0139 ± 0.0003*** ** |
| | 0.3 | **0.0146 ± 0.001*** ** |
| **Comp. 3** | 0.1 | **0.0137 ± 0.0003*** ** |
| | 0.3 | **0.0144 ± 0.0005*** ** |
| **Comp. 4** | 0.1 | **0.0134 ± 0.001** ** |
| | 0.3 | **0.0138 ± 0.0007*** ** |
| **Comp. 5** | 0.1 | **0.0132 ± 0.0008* ** |
| | 0.3 | **0.0135 ± 0.0011** ** |
| **Comp. 6** | 0.1 | 0.0128 ± 0.0007 |
| | 0.3 | 0.0129 ± 0.0007 |
| **Comp. 7** | 0.1 | 0.0131 ± 0.0013 |
| | 0.3 | **0.0139 ± 0.001* ** |
| **Comp. 8** | 0.1 | 0.0131 ± 0.0009 |
| | 0.3 | **0.0138 ± 0.0015* ** |

An asterisk denotes the significant differences between control and cells treated with glucose, metformin or studied compounds. * $p < 0.05$; ** $p < 0.01$; *** $p < 0.001$.

**Table S2** The effects of sulfonamides **1–5** on HUVEC cells growth. The results express the percentage of cell viability at the compound concentration of 0.06 and 0.3 mM in comparison to control samples. The results are presented as mean ± SD (n = 8).

| | *HUVEC cells growth* | | |
|---|---|---|---|
| *Compound* | **CTR** | **0.06 mM** | **0.3 mM** |
| ***1*** | 100.00 ± 6.32 | **76.35 ± 9.19*** ** | **71.04 ± 8.45*** ** |
| ***2*** | 101.87 ± 2.71 | **75.91 ± 5.85*** ** | **71.48 ± 3.12*** ** |
| ***3*** | 100.00 ± 5.85 | **77. 89 ± 4.10*** ** | **75. 85 ± 11.77*** ** |
| ***4*** | 100. 00 ± 5.85 | **81.54 ± 9.49*** ** | **73.93 ± 5.30*** ** |
| ***5*** | 103.77 ± 8.09 | **84. 79 ± 5.67*** ** | **77.85 ± 6.65*** ** |

**Table S3.** The effects of compounds **1** – **8** on the integrity of HUVECs analyzed in the RTCA-DP system.

| Compound | Concentration | 3 hours | 6 hours | 12 hours | 24 hours | 36 hours |
|---|---|---|---|---|---|---|
| CTR | - | 1.181 ± 0.124 | 1.124 ± 0.051 | 1.168 ± 0.094 | 1.111 ± 0.166 | 0.756 ± 0.192 |
| 1 | 0.3 mM | 0.832 ± 0.091** | 0.981 ± 0.085 | 1.177 ± 0.149 | 1.207 ± 0.149** | 0.875 ± 0.090 |
| | 1.0 mM | 0.440 ± 0.032*** | 0.676 ± 0.062*** | 0.791 ± 0.067*** | 0.940 ± 0.077*** | 0.858 ± 0.054 |
| 2 | 0.3 mM | 0.608 ± 0.038*** | 0.693 ± 0.022*** | 0.676 ± 0.047*** | 0.522 ± 0.030*** | 0.761 ± 0.008*** |
| | 1.0 mM | 0.652 ± 0.006*** | 0.706 ± 0.007*** | 0.719 ± 0.005*** | 0.731 ± 0.002*** | 0.379 ± 0.032* |
| 3 | 0.3 mM | 0.680 ± 0.083*** | 0.837 ± 0.213** | 0.927 ± 0.212*** | 0.969 ± 0.156*** | 0.714 ± 0.035** |
| | 1.0 mM | 0.233 ± 0.039*** | 0.554 ± 0.047*** | 0.616 ± 0.042*** | 0.748 ± 0.061*** | 0.694 ± 0.054*** |
| 4 | 0.3 mM | 1.026 ± 0.008** | 0.977 ± 0.004** | 0.940 ± 0.010** | 0.881 ± 0.013** | 0.777 ± 0.021*** |
| | 1.0 mM | 0.592 ± 0.031*** | 0.583 ± 0.029*** | 0.515 ± 0.038*** | 0.494 ± 0.024*** | 0.339 ± 0.030*** |
| 5 | 0.3 mM | 1.146 ± 0.108 | 1.036 ± 0.017* | 0.746 ± 0.010*** | 0.830 ± 0.001*** | 0.690 ± 0.017* |
| | 1.0 mM | 0.438 ± 0.042*** | 0.501 ± 0.027*** | 0.513 ± 0.050*** | 0.557 ± 0.008*** | 0.275 ± 0.021*** |
| 6 | 0.3 mM | 0.317 ± 0.050*** | 0.424 ± 0.053*** | 0.388 ± 0.07*** | 0.280 ± 0.018*** | 0.111 ± 0.026*** |
| | 1.0 mM | 0.010 ± 0.003*** | 0.016 ± 0.005*** | 0.019 ± 0.005*** | 0.013 ± 0.003*** | 0.009 ± 0.003*** |
| 7 | 0.3 mM | 0.283 ± 0.050*** | 0.367 ± 0.027*** | 0.333 ± 0.030*** | 0.255 ± 0.26*** | 0.094 ± 0.014*** |
| | 1.0 mM | 0.013 ± 0.004*** | 0.018 ± 0.001*** | 0.020 ± 0.001*** | 0.016 ± 0.002*** | 0.013 ± 0.003*** |
| 8 | 0.3 mM | 0.312 ± 0.087*** | 0.461 ± 0.015*** | 0.416 ± 0.062*** | 0.321 ± 0.038*** | 0.198 ± 0.055*** |
| | 1.0 mM | 0.006 ± 0.004*** | 0.029 ± 0.006*** | 0.021 ± 0.004*** | 0.001 ± 0.001*** | 0.001 ± 0.001*** |

The results are presented as mean ± SD, n = 4 – 6. The table includes the calculated normalized Cell Index values (nCI) for control and compounds **1** – **8** after selected time points (3 – 36 h) of incubation. An asterisk denotes the statistically significant difference between sample treated with compounds and controls ample (* $p < 0.05$; ** $p < 0.01$; *** $p < 0.001$).

**Table S4.** The effects of metformin and compounds **1** - **5** on the migration of HUVECs analyzed in the JulieStage system. The results are presented as a wound width [μm].

| Compound | Concentration | Start [μm] | 6 hours [μm] | 12 hours [μm] | 18 hours [μm] | 24 hours [μm] |
|---|---|---|---|---|---|---|
| **CTR** | - | 778.2 ± 189.9 | 619.2 ± 116.0 | 526.3 ± 120.4 | 383.2 ± 54.4 | 272.4 ± 42.2 |
| **1** | 0.3 mM | 605.8 ± 38.63 | 491.9 ± 31.7 | 421.2 ± 5.9 | 365.2 ± 35.8 | 336.7 ± 39.0 |
| | 1.0 mM | 589.1 ± 36.3 | 461.9 ± 33.4* | 375.1 ± 29.9 | 344.4 ± 32.8 | 337.5 ± 47.3 |
| **2** | 0.3 mM | 637.4 ± 116.3 | 493.8 ± 133.1 | 442.4 ± 142.9 | 351.4 ± 82.6 | 328.6 ± 56.9 |
| | 1.0 mM | 712.4 ± 56.5 | 590.8 ± 40.2 | 541.5 ± 99.2 | 453.1 ± 106.1 | 357.2 ± 68.1 |
| **3** | 0.3 mM | 675.1 ± 94.6 | 606.7 ± 149.4 | 486.0 ± 131.2 | 441.9 ± 99.5 | 395.9 ± 104.7 |
| | 1.0 mM | 609.3 ± 73.0 | 494.6 ± 106.9 | 414.1 ± 103.5 | 385.2 ± 94.5 | 331.3 ± 62.5 |
| **4** | 0.3 mM | 681.2 ± 226.7 | 509.0 ± 188.7 | 418.6 ± 140.4 | 326.8 ± 146.6 | 271.1 ± 84.6 |
| | 1.0 mM | 659.9 ± 139.7 | 579.6 ± 127.6 | 498.9 ± 146.5 | 406.6 ± 145.3 | 352.5 ± 112.2 |
| **5** | 0.3 mM | 606.2 ± 55.1 | 450.7 ± 44.6 | 388.3 ± 26.8 | 318.3 ± 16.7 | 277.1 ± 50.3 |
| | 1.0 mM | 609.0 ± 127.2 | 541.5 ± 78.2 | 497.4 ± 36.8 | 452.5 ± 46.3 | 454.5 ± 62.0* |
| **MET** | 0.3 mM | 728.6 ± 62.5 | 570.1 ± 119.9 | 467.8 ± 106.5 | 412.1 ± 121.8 | 405.0 ± 120.7 |
| | 1.0 mM | 670.4 ± 29.2 | 565.4 ± 31.5 | 495.7 ± 20.0 | 482.7 ± 10.0 | 419.8 ± 42.3 |

The results are presented as mean ± SD, n = 4 – 8; (* $p < 0.05$).

**Table S5.** Effects of biguanides **1** – **5** on the activity of factor X (data are presented as mean ± SD; n = 4–5). The asterisk denotes a statistically significant difference between the samples treated with compounds and control; * $p < 0.05$, ** $p < 0.01$.

| | **Factor X activity [%]** | | | | |
|---|---|---|---|---|---|
| **Compound** | **Control** | **Control with solvent** | **Concentration** | | |
| | | | 0.3 mM | 0.6 mM | 1.5 mM |
| **1** | 109.20 ± 5.45 | 106.20 ± 1.79 | 107.60 ± 4.62 | 104.20 ± 4.97 | 97.40 ± 8.05** |
| **2** | 109.20 ± 5.45 | 106.20 ± 1.79 | 107.40 ± 6.19 | 104.00 ± 5.34 | 107.00 ± 4.00 |
| **3** | 109.20 ± 5.45 | 106.20 ± 1.79 | 105.60 ± 2.30 | 103.40 ± 7.23 | 104.20 ± 8.14 |
| **4** | 109.20 ± 5.45 | 106.20 ± 1.79 | 102.00 ± 5.15 | 100.80 ± 5.17 | 98.80 ± 11.39 |
| **5** | 109.20 ± 5.45 | 106.20 ± 1.79 | 109.80 ± 3.35 | 106.20 ± 3.27 | 102.20 ± 3.77* |

**Table S6.** Effects of selected biguanides **1** – **5** on the activity of AT III (data are presented as mean ± SD; n = 4). The asterisk denotes a statistically significant difference between the samples treated with compounds and control; * $p < 0.05$, ** $p < 0.01$.

| **Compound** | **Control** | **Concentration** | | | |
|---|---|---|---|---|---|
| | | 0.06 mM | 0.3 mM | 0.6 mM | 1.5 mM |
| **1** | 109.75 ± 3.86 | 110.25 ± 8.34 | 115.25 ± 6.18 | 120.00 ± 2.71** | 123.00 ± 5.2** |
| **2** | | 121.00 ± 4.90 | 127.75 ± 10.44 | 125.75 ± 10.87 | 129.00 ± 11.46 |
| **3** | | 113.00 ± 10.13 | 117.25 ± 5.50 | 131.75 ± 6.65* | 136.75 ± 11.32* |
| **4** | | 113.00 ± 1.41 | 118.00 ± 5.29 | 123.50 ± 8.06* | 127.25 ± 10.37* |
| **5** | | 115.00 ± 12.81 | 114.50 ± 8.89 | 118.50 ± 8.50 | 128.75 ± 5.85** |

**Table S7.** The effects of synthesized sulfonamide derivatives of metformin **1 – 5** on the kinetic parameters of clot formation and fibrinolysis (CL-test).

| | | Parameters | | | | | | | | | | | | |
|---|---|---|---|---|---|---|---|---|---|---|---|---|---|---|
| **Compound** | **Concentr. [mM]** | **Tt [s]** | **Fmax [%T]** | **Tf [s]** | **Fvo [%T/min]** | **Tc [s]** | **Lmax [%T]** | **Tl [s]** | **Lvo [%T/min]** | **Sf [%T x min]** | **Sc [%T x min]** | **Sl [%T x min]** | **S [%T x min]** | **T [s]** |
| **1** | 0 (CTR) | 21.76 ± 4.24 | 65.79 ± 7.55 | 56.67 ± 7.10 | 203.00 ± 33.04 | 274.43 ± 69.69 | 65.23 ± 7.32 | 228.29 ± 65.51 | 18.74 ± 5.35 | 43.87 ± 6.67 | 293.56 ± 81.99 | 140.49 ± 42.97 | 477.92 ± 128.92 | 581.14 ± 137.78 |
| | 0.06 | **18.97 ± 3.08*** | **73.71 ± 5.89**** | 58.61 ± 9.61 | 230.57 ± 34.38 | 324.86 ± 130.79 | 70.23 ± 10.26 | 253.57 ± 104.13 | 21.40 ± 6.16 | **50.97 ± 7.65**** | 389.25 ± 160.80 | **193.67 ± 107.75*** | **633.89 ± 269.28*** | 656.01 ± 236.60 |
| | 0.3 | **16.81 ± 3.05**** | **71.97 ± 6.78**** | 61.36 ± 11.85 | 227.86 ± 37.81 | **347.71 ± 124.84*** | 69.09 ± 10.62 | **260.57 ± 95.60*** | 18.53 ± 7.15 | **52.45 ± 10.08**** | **407.13 ± 154.90*** | **186.37 ± 104.97**** | **645.95 ± 264.51**** | **686.46 ± 226.50*** |
| | 0.6 | **16.75 ± 4.87**** | **70.77 ± 5.62*** | **63.63 ± 6.31*** | 217.71 ± 37.42 | **338.00 ± 114.76**** | 69.47 ± 6.67 | **267.71 ± 99.31*** | 18.64 ± 4.38 | **53.53 ± 5.26***** | **388.20 ± 131.19*** | 178.97 ± 84.17 | **620.70 ± 216.54*** | **686.09 ± 216.29*** |
| | 1.5 | 21.60 ± 6.05 | **70.21 ± 3.38*** | **64.21 ± 9.03*** | 193.57 ± 47.31 | 344.29 ± 149.70 | 66.90 ± 8.86 | 236.57 ± 80.20 | 20.23 ± 6.46 | **52.36 ± 6.58*** | 389.89 ± 172.66 | 159.20 ± 87.92 | 602.52 ± 263.77 | 666.67 ± 234.05 |
| **2** | 0 (CTR) | 19.64 ± 4.14 | 60.09 ± 6.33 | 51.63 ± 6.91 | 190.14 ± 20.85 | 272.29 ± 56.91 | 60.49 ± 6.12 | 223.14 ± 47.05 | 19.41 ± 2.85 | 36.43 ± 7.98 | 267.76 ± 72.76 | 122.83 ± 33.42 | 427.01 ± 111.41 | 566.70 ± 105.96 |
| | 0.06 | **16.86 ± 4.24*** | **66.77 ± 5.68*** | **56.56 ± 8.57*** | 203.43 ± 22.00 | **296.57 ± 72.31*** | **67.40 ± 5.38**** | 240.14 ± 65.11 | 22.29 ± 4.30 | **44.40 ± 9.07***** | **323.20 ± 94.05**** | **148.45 ± 47.41*** | **516.05 ± 146.55**** | 610.13 ± 145.50 |
| | 0.3 | 17.54 ± 5.79 | **68.99 ± 5.55*** | 59.24 ± 16.26 | 194.00 ± 48.64 | **309.14 ± 68.74**** | **69.49 ± 5.34*** | 252.00 ± 73.00 | 19.83 ± 6.57 | **46.00 ± 9.83***** | **344.01 ± 74.34***** | **153.63 ± 43.80**** | **543.64 ± 122.37***** | **637.93 ± 155.22*** |
| | 0.6 | 17.57 ± 3.74 | 67.40 ± 9.57 | 55.80 ± 11.60 | 214.14 ± 50.85 | **319.00 ± 63.63**** | 68.19 ± 9.82 | 247.43 ± 74.21 | 20.37 ± 7.03 | **44.34 ± 10.50*** | **344.82 ± 65.62*** | 143.77 ± 40.07 | **532.93 ± 111.10*** | **639.80 ± 148.09*** |
| | 1.5 | **24.37 ± 7.41*** | 61.57 ± 7.24 | 61.21 ± 14.65 | 177.43 ± 34.91 | **351.43 ± 96.53**** | 62.14 ± 6.63 | 249.14 ± 80.94 | 20.07 ± 6.59 | **44.31 ± 12.08*** | **346.35 ± 92.32**** | 133.78 ± 47.19 | **524.43 ± 146.77*** | **686.16 ± 190.86*** |
| **3** | 0 (CTR) | 18.82 ± 3.69 | 61.83 ± 2.64 | 47.35 ± 9.66 | 187.33 ± 40.60 | 246.17 ± 36.41 | 66.15 ± 9.79 | 241.50 ± 92.10 | 19.25 ± 5.44 | 33.50 ± 9.79 | 249.97 ± 34.38 | 137.96 ± 53.57 | 421.43 ± 60.96 | 553.83 ± 88.64 |
| | 0.06 | 16.68 ± 3.26 | 65.40 ± 9.39 | **57.45 ± 8.71*** | 208.17 ± 44.80 | 279.50 ± 46.51 | 71.38 ± 8.36 | 235.33 ± 48.33 | 20.38 ± 5.24 | **48.52 ± 10.81*** | **314.15 ± 49.14*** | 156.16 ± 30.07 | **521.91 ± 66.81***** | 588.97 ± 87.54 |
| | 0.3 | 16.23 ± 4.22 | **68.78 ± 4.04**** | 55.13 ± 7.55 | 214.17 ± 32.05 | **299.50 ± 38.60**** | 70.15 ± 3.87 | 229.17 ± 48.85 | 22.88 ± 8.98 | **44.41 ± 6.00*** | **335.21 ± 50.51**** | 142.20 ± 37.46 | **521.82 ± 88.83**** | 600.03 ± 91.80 |
| | 0.6 | 17.17 ± 3.17 | **66.60 ± 4.00**** | 54.73 ± 5.88 | 211.50 ± 22.76 | **299.50 ± 40.90*** | 67.55 ± 4.50 | 234.83 ± 42.86 | 23.53 ± 6.67 | 42.40 ± 3.94 | **323.11 ± 42.53**** | 137.48 ± 23.47 | **502.99 ± 64.69***** | 606.23 ± 87.70 |
| | 1.5 | 18.15 ± 4.30 | 63.60 ± 5.36 | **62.35 ± 6.00*** | 199.50 ± 18.07 | **355.50 ± 66.77**** | 63.92 ± 5.38 | 253.17 ± 62.51 | 23.50 ± 13.77 | **47.68 ± 7.07*** | **368.60 ± 84.14*** | 143.17 ± 38.79 | **559.45 ± 127.25*** | **689.17 ± 134.48**** |
| **4** | 0 (CTR) | 21.02 ± 2.10 | 66.46 ± 3.97 | 50.98 ± 3.76 | 209.80 ± 18.21 | 243.00 ± 44.06 | 66.64 ± 3.91 | 185.80 ± 33.86 | 22.86 ± 2.93 | 39.76 ± 4.23 | 262.61 ± 52.40 | 111.94 ± 19.65 | 414.31 ± 74.15 | 500.80 ± 81.25 |
| | 0.06 | 17.90 ± 1.55 | 70.78 ± 2.99 | 56.08 ± 4.04 | 218.80 ± 18.83 | 254.40 ± 42.82 | 70.60 ± 2.68 | 192.40 ± 34.02 | 22.84 ± 2.84 | **46.76 ± 4.65**** | 293.55 ± 59.85 | 128.24 ± 33.57 | 468.55 ± 95.54 | 520.78 ± 76.54 |
| | 0.3 | 18.10 ± 1.60 | 67.68 ± 2.97 | 53.62 ± 5.86 | 213.40 ± 35.45 | 267.20 ± 50.90 | 68.22 ± 3.35 | 199.60 ± 31.48 | 23.44 ± 1.16 | 42.11 ± 3.94 | 295.28 ± 65.79 | 121.04 ± 21.89 | 458.43 ± 88.55 | 538.52 ± 81.13 |
| | 0.6 | 19.26 ± 3.17 | 67.36 ± 4.41 | **57.16 ± 4.14*** | 204.60 ± 17.01 | **271.80 ± 34.83*** | 67.86 ± 4.50 | 192.60 ± 30.81 | 23.86 ± 5.29 | 45.49 ± 5.26 | **296.64 ± 39.28*** | 114.75 ± 16.61 | **456.88 ± 57.50*** | **540.82 ± 63.83*** |
| | 1.5 | 21.50 ± 1.37 | **58.40 ± 4.18**** | **63.84 ± 5.82**** | **169.80 ± 13.83*** | **281.40 ± 41.02*** | 59.92 ± 6.34 | 195.00 ± 35.57 | 22.96 ± 6.37 | 44.23 ± 5.10 | 267.39 ± 29.91 | **101.06 ± 16.22*** | 410.69 ± 41.73 | **561.74 ± 73.93*** |
| **5** | 0 (CTR) | 20.02 ± 1.26 | 66.28 ± 7.48 | 52.16 ± 3.51 | 217.20 ± 37.39 | 280.80 ± 29.23 | 66.46 ± 5.58 | 231.40 ± 20.77 | 24.12 ± 6.46 | 40.79 ± 6.01 | 301.89 ± 45.53 | 146.24 ± 30.82 | 488.91 ± 75.48 | 584.38 ± 46.77 |
| | 0.06 | **15.90 ± 1.94**** | 70.66 ± 8.17 | 56.96 ± 4.97 | 225.00 ± 34.12 | **309.60 ± 33.97**** | 72.16 ± 7.80 | 254.40 ± 11.06 | 20.20 ± 5.10 | **47.54 ± 4.13*** | **357.76 ± 75.93*** | 159.03 ± 25.98 | 564.32 ± 102.00 | **636.86 ± 37.88*** |
| | 0.3 | **17.00 ± 2.17*** | 70.94 ± 4.68 | 57.26 ± 5.37 | 227.60 ± 13.30 | **331.40 ± 24.11**** | 66.96 ± 11.55 | 244.00 ± 21.58 | 20.18 ± 3.47 | **48.43 ± 6.81**** | **383.11 ± 50.30***** | **169.87 ± 46.38*** | **601.41 ± 90.65***** | **649.66 ± 38.22**** |
| | 0.6 | **16.22 ± 1.62**** | 69.22 ± 6.62 | 56.60 ± 7.39 | 219.60 ± 31.60 | 348.60 ± 50.47 | 70.12 ± 6.38 | 257.00 ± 49.10 | 22.48 ± 6.56 | 46.55 ± 8.60 | **393.45 ± 83.29*** | 158.15 ± 42.23 | 598.15 ± 132.27 | 678.42 ± 105.62 |
| | 1.5 | 17.44 ± 2.25 | 67.02 ± 7.66 | **61.12 ± 6.91*** | 207.80 ± 42.70 | 336.00 ± 40.02 | 69.08 ± 8.20 | 252.80 ± 32.96 | 18.28 ± 3.58 | **48.35 ± 4.83*** | **366.87 ± 74.64*** | 146.13 ± 30.60 | 561.34 ± 104.05 | 667.36 ± 65.44 |

The results are presented as mean ± standard deviation, n = 5 – 7. The asterisk denotes a statistically significant difference between the samples treated with compounds and control; * $p < 0.05$, ** $p < 0.01$, *** $p < 0.001$. Tt—thrombin time, Fmax—maximum clotting, Tf— plasma clotting time, Fvo—initial plasma clotting velocity, Tc—clot stabilization time, Lmax — maximum lysis, Tl — fibrynolisis time, Lvo—initial clot fibrinolysis velocity, Sf—area under the clot formation curve, Sc—area under the curve of a stable clot formation, Sr — area under the fibrinolysis curve, S — overall potential of clot formation and fibrinolysis, T—total time of the process of clot formation and fibrinolysis.

**Table S8.** The effects of synthesized sulfonamide derivatives of metformin **1** – **5** on the process of coagulation after generation of endogenous thrombin (Coagulation Assay).

| Parameters | | | | | | | | |
|---|---|---|---|---|---|---|---|---|
| **Compound** | **Concentr. [mM]** | **TGt [s]** | **Fmax [%T]** | **Tf [s]** | **Fvo [%T/min]** | **Sf [%T x min]** | **Sc [%T x min]** | **S [%T x min]** |
| **1** | 0 (CTR) | 148.17 ± 34.31 | 77.82 ± 11.86 | 115.73 ± 40.18 | 128.05 ± 31.13 | 103.38 ± 24.78 | 533.26 ± 112.97 | 1493.77 ± 236.20 |
| | 0.06 | 161.33 ± 35.27 | 76.30 ± 7.79 | **155.67 ± 45.29*** | 97.22 ± 36.13 | **140.16 ± 31.01*** | 458.27 ± 113.77 | 1449.09 ± 154.09 |
| | 0.3 | 162.33 ± 37.96 | 82.67 ± 4.79 | 109.63 ± 40.06 | 117.65 ± 36.38 | 104.53 ± 38.44 | 544.47 ± 81.13 | 1586.01 ± 103.04 |
| | 0.6 | 176.83 ± 57.02 | 75.62 ± 9.88 | 138.77 ± 59.29 | **96.52 ± 20.91*** | 119.01 ± 48.07 | 444.40 ± 91.71 | 1442.42 ± 198.39 |
| | 1.5 | 172.00 ± 43.56 | 74.15 ± 7.83 | 135.33 ± 75.79 | 107.77 ± 35.80 | 114.89 ± 64.82 | 452.95 ± 102.22 | 1435.77 ± 143.80 |
| **2** | 0 (CTR) | 130.60 ± 22.66 | 84.52 ± 5.26 | 93.72 ± 17.54 | 133.05 ± 34.97 | 87.96 ± 15.40 | 629.50 ± 62.21 | 1627.12 ± 106.89 |
| | 0.06 | 151.33 ± 26.37 | 85.20 ± 6.52 | 98.28 ± 33.46 | 124.35 ± 37.66 | 91.44 ± 30.65 | 562.66 ± 156.02 | 1638.19 ± 135.59 |
| | 0.3 | 135.67 ± 25.90 | 85.12 ± 5.67 | 85.08 ± 25.10 | 142.17 ± 46.90 | 79.63 ± 20.32 | 602.85 ± 84.63 | 1641.98 ± 118.29 |
| | 0.6 | 153.33 ± 21.05 | 83.60 ± 6.72 | 91.58 ± 19.43 | 140.83 ± 37.45 | 88.00 ± 18.58 | 583.07 ± 90.13 | 1611.84 ± 135.58 |
| | 1.5 | **170.50 ± 31.73*** | **76.35 ± 9.75*** | 101.45 ± 38.76 | 120.32 ± 48.40 | 87.66 ± 44.66 | 511.33 ± 178.11 | **1467.86 ± 181.79**** |
| **3** | 0 (CTR) | 157.2 ± 18.14 | 82.76 ± 8.29 | 109.20 ± 43.90 | 125.50 ± 34.76 | 98.32 ± 34.87 | 550.86 ± 105.32 | 1551.16 ± 128.03 |
| | 0.06 | 155.00 ± 20.58 | 84.78 ± 5.57 | 108.30 ± 35.15 | 108.56 ± 28.63 | 102.35 ± 29.66 | 586.27 ± 95.97 | 1628.36 ± 118.58 |
| | 0.3 | 139.60 ± 7.44 | 82.66 ± 7.51 | 108.58 ± 33.64 | 113.42 ± 23.98 | 100.51 ± 28.00 | 766.41 ± 394.94 | 1580.91 ± 150.03 |
| | 0.6 | 158.40 ± 13.03 | 82.44 ± 5.61 | 112.94 ± 35.34 | 127.78 ± 40.82 | 107.67 ± 29.19 | 560.45 ± 102.47 | 1580.02 ± 119.57 |
| | 1.5 | 165.40 ± 28.25 | 77.50 ± 8.20 | 93.42 ± 26.49 | 135.98 ± 31.06 | 83.28 ± 17.34 | 537.91 ± 99.63 | 1494.31 ± 163.29 |
| **4** | 0 (CTR) | 154.14 ± 23.86 | 74.67 ± 9.74 | 112.94 ± 32.85 | 102.23 ± 31.19 | 94.02 ± 21.11 | 510.67 ± 100.18 | 1429.48 ± 196.15 |
| | 0.06 | 161.71±39.45 | 79.24±7.60 | 129.97±38.90 | 96.99±11.47 | 119.40±39.08 | 515.40±98.15 | 1519.15±151.73 |
| | 0.3 | 181.86±60.74 | 80.94±9.46 | **170.66±58.09**** | **83.81±30.97**** | **153.41±43.56***** | 458.09±90.33 | 1521.04±185.84 |
| | 0.6 | **219.43±38.58*** | 82.30±8.88 | **146.74±41.02*** | **65.83±16.80*** | **122.37±37.64*** | **421.14±95.69*** | 1556.25±169.91 |
| | 1.5 | 202.71±57.08 | 73.63±11.18 | 142.07±47.27 | 74.69±22.75 | 114.15±40.37 | **412.12±85.19*** | 1385.35±201.92 |
| **5** | 0 (CTR) | 148.71 ± 28.25 | 83.49 ± 4.87 | 83.41 ± 17.23 | 149.00 ± 40.89 | 78.61 ± 11.72 | 610.13 ± 83.82 | 1612.34 ± 102.21 |
| | 0.06 | **160.57 ± 14.68*** | 83.37 ± 7.62 | 123.04 ± 59.00 | **103.67 ± 43.45*** | 110.80 ± 46.90 | 534.01 ± 122.74 | 1577.51 ± 142.35 |
| | 0.3 | **193.29 ± 39.67*** | 84.50 ± 4.38 | 119.30 ± 57.99 | **110.86 ± 46.57*** | 110.58 ± 52.91 | **505.31 ± 135.62*** | 1613.22 ± 106.60 |
| | 0.6 | 196.71 ± 54.37 | **78.74 ± 8.04*** | **127.66 ± 35.79*** | **93.69 ± 26.75**** | 115.19 ± 39.76 | **462.63 ± 86.60*** | 1427.86 ± 277.45 |
| | 1.5 | 168.67 ± 35.31 | **73.12 ± 6.99**** | **121.73 ± 32.28*** | **110.63 ± 31.15*** | 105.87 ± 31.97 | **490.51± 52.81**** | **1399.07 ± 139.67**** |

The results are presented as mean ± standard deviation, n = 5 – 7. The asterisk denotes a statistically significant difference between the samples treated with compounds and control; * $p < 0.05$, ** $p < 0.01$,*** $p < 0.001$. TGt—thrombin generation time, Fmax—maximum clotting, Tf— plasma clotting time, Fvo—initial plasma clotting velocity, Sf—area under the clot formation curve, Sc—area under the curve of a stable clot formation, S — overall potential of clot formation.
